# Catalytic Depolymerization of Polyester Waste via Zinc Oxide‐Decorated Silica

**DOI:** 10.1002/advs.202514922

**Published:** 2025-10-27

**Authors:** Jingjing Cao, Huaxing Liang, Wei Chen, Jiachen Dong, Zhiguang Li, Shaohai Fu

**Affiliations:** ^1^ Key Laboratory of Special Protective Textiles Ministry of Education College of Textile Science and Engineering Jiangnan University Wuxi 214122 China; ^2^ Department of Environmental Science and Engineering University of Science and Technology of China Hefei 230026 China; ^3^ Key Laboratory of Green and Low‐Carbon Processing Technology for Fibers and Textiles Binzhou 256600 China

**Keywords:** circular economy, defect catalyst, glycolysis mechanism, life cycle assessment, polyester waste management

## Abstract

The aromatic units in the polyester materials are chemically robust, thermally stable, and widely used as engineering plastics. However, their chemical recycling remains a significant challenge. Herein, a zinc oxide‐decorated silica (ZnO/SiO_2_) catalyst is reported for the efficient chemical depolymerization of polyester into value‐added bis(2‐hydroxyethyl) terephthalate (BHET). The process affords BHET in 92.5% yield and 99% purity, with the yield remaining at 90% even after five cycles and an extended reaction time of 2 h. The strategy leverages surface defects on ZnO/SiO_2_ to activate ethylene glycol and polarize ester carbonyls, thereby facilitating ester bond cleavage. A wide range of polyester waste plastics (i.e., mixed plastics, textiles, and packaging materials) are effectively depolymerized, underscoring the broad applicability of the catalytic system. Life cycle assessment demonstrates the viability of the recycling approach, achieving 235% energy savings and 104% reduction in greenhouse gas emissions compared to petroleum‐based production of virgin BHET processes. Moreover, utilizing textile scrap further leads to a five‐fold reduction in minimum selling price. This work offers a sustainable solution for managing PET waste and to realizing the circular economy.

## Graphical abstract

Plastics recycling into value‐added products has attracted much interests, but generally comes at a high energy cost. Here, the authors present a zinc oxide‐decorated silica catalyst for the efficient depolymerization of polyethylene terephthalate (PET) into bis(2‐hydroxyethyl)terephthalate (BHET) and ethylene glycol (EG). This strategy demonstrates reduced energy demand, techno‐economic feasibility, and lower environmental impact compared to conventional chemical recycling approaches.

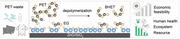

## 1 Introduction

Polyester‐based plastics, typically synthesized via the condensation of polyols and polyacids,^[^ ^1^ ^]^ account for ≈15% of total plastic waste. The aromatic units in the petroleum‐based polyester backbone impart rigidity, mechanical strength, and thermal stability, while the ethylene glycol segments contribute flexibility.^[^ ^2^, ^3^, ^4^, ^5^ ^]^ These structural characteristics endow polyesters with excellent physical properties. However, they also pose environmental challenges and resource losses.^[^ ^6^, ^7^, ^8^ ^]^ Chemical depolymerization of polyesters into monomers or value‐added chemicals is widely regarded as a promising strategy for plastic circularity.^[^ ^9^, ^10^, ^11^, ^12^ ^]^ Central to this approach is the development of efficient homogeneous and heterogeneous catalytic systems. Owing to their facile separation from reaction mixtures and compatibility with industrial processes, heterogeneous catalysts have attracted increasing attention.

To date, a variety of heterogeneous catalysts (e.g., metal oxides, spinel, metal–organic frameworks, etc.) have been developed for polyester recycling.^[^ ^13^, ^14^, ^15^, ^16^ ^]^ Their intrinsic Lewis acidity, structural tunability, and robustness make them ideal candidates.^[^ ^17^, ^18^, ^19^, ^20^ ^]^ However, conventional design strategies typically focus on modulating surface catalytic sites, often neglecting broader structural optimization. This limits the accessibility of active sites, especially in reactions involving high‐molecular‐weight, viscous polyester substrates, thus hindering mass transfer and lowering catalytic efficiency. Moreover, when the oligomeric intermediates generated during polyester depolymerization fail to undergo timely bond cleavage, they tend to accumulate on the catalyst surface, leading to catalyst deactivation.

Emerging strategies aimed at enhancing catalytic activity (such as tuning catalyst particle size, leveraging photothermal effects, and employing porous materials, etc.), which can significantly improve catalyst dispersibility in the reaction medium.^[^ ^21^, ^22^, ^23^, ^24^, ^25^, ^26^ ^]^ These approaches play a critical role in activating catalytic sites and facilitating the cleavage of ester bonds in polyester plastics. Chen and co‐workers employed Zn^2+^ ions in the Zn‐MCM‐41 catalyst as Lewis's acid centers to coordinate with the carbonyl oxygen of PET, thereby promoting ester bond cleavage.^[^ ^27^ ^]^ However, these Zn^2+^ sites are confined within the mesoporous channels of the molecular sieve, significantly limiting mass transfer between the catalyst, polyester, and solvent. Chen and co‐workers designed a silica‐coated metal–organic frameworks (MOFs) photothermal catalyst and optimized its activity by adjusting the silica shell thickness.^[^ ^28^ ^]^ While the silica layer reduced thermal radiation loss and enhanced localized photothermal effects, it also impeded mass transfer between active sites and reactants, ultimately lowering catalytic efficiency. Inspired by the silicon–copper “contact” interface catalysis used in industrial chlorosilane production, Ding Kunlun and colleagues developed a method for PET depolymerization via methanolysis by introducing a trace heterogeneous catalyst layer directly onto the PET surface.^[^ ^29^ ^]^ In this system, electrostatically adsorbed Zn^2+^ played a key role in enabling efficient ester exchange and depolymerization. Photothermally active heterogeneous catalysts, such as CoMn_2_O_4_ and grain boundary–rich CeO_2_, have recently been explored for PET glycolysis.^[^ ^30^, ^31^, ^32^, ^33^, ^34^ ^]^ These multifunctional catalysts exhibit high reactivity, excellent stability, and efficient conversion under low light intensities. More broadly, the construction of defect‐rich heterogeneous metal oxide catalysts has proven effective, as the unsaturated coordination sites can interact with polyester carbonyl groups, thereby facilitating nucleophilic addition–elimination reactions for ester bond cleavage. Despite their potential for chemical recycling, progress remains hampered by interfacial mass transfer limitations and poorly understood catalytic mechanisms at heterogeneous interfaces. Therefore, the rational design of defect‐rich heterogeneous catalysts is crucial for enhancing product selectivity, improving catalyst robustness, and overcoming the transport limitations across complex solid–liquid interfaces.

Here, we developed a zinc oxide‐decorated silica (ZnO/SiO_2_) catalyst by anchoring ZnO nanoparticles onto silica surfaces, forming isolated and defect‐rich sites. This strategy enables efficient depolymerization of diverse polyester wastes, including beverage bottles, textiles, packaging materials, and mixed plastic streams, into their corresponding monomers. The catalyst delivers outstanding performance, achieving 100% PET conversion and over 92.5% yield of bis(2‐hydroxyethyl) terephthalate (BHET), along with remarkable reusability. These results are attributed to the high density and stability of surface defect sites on the ZnO/SiO_2_ interface. In situ FTIR spectroscopy reveals that the abundant defects efficiently activate the O─H bond in ethylene glycol and the C═O bond in polyester, clarifying the reaction pathway and intermediate species involved. Life cycle assessment (LCA) demonstrates considerable environmental advantages of this method over terephthalic acid (TPA) synthesis routes, including reductions in human health impact, resource consumption, and ecosystem burden. Moreover, a techno‐economic analysis (TEA) confirmed the economic feasibility of the process, with a minimum selling price (MSP) of 233.6 $ ton^−1^, which is much lower than that of TPA synthesis routes (1435.3 $ ton^−1^). This work provides a sustainable and economically viable strategy for polyester waste valorization.

## 2 Results and Discussion

### 2.1 Synthesis and Characterization of ZnO/SiO_2_

**Figure 1:**
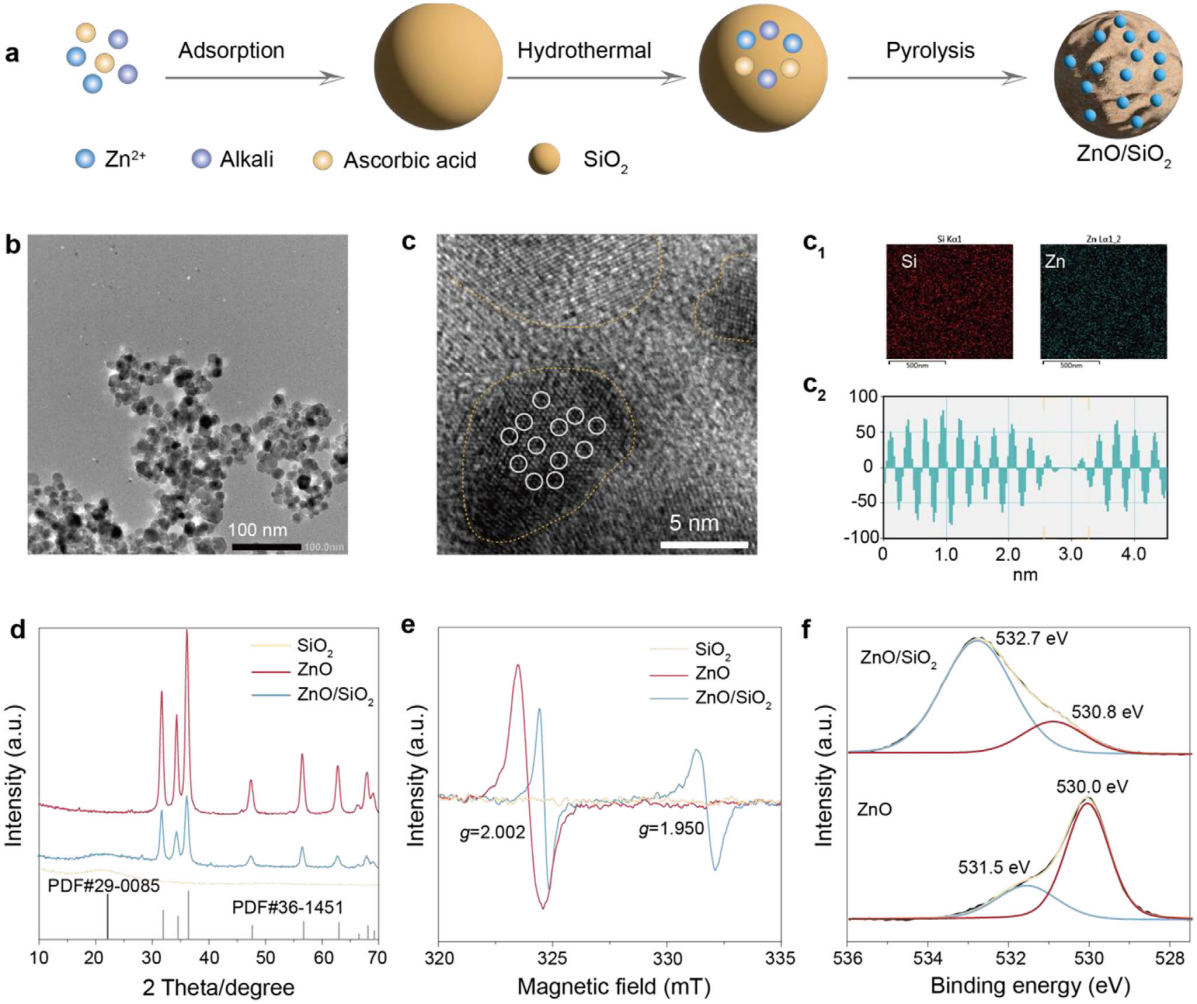
Synthesis and characterization of materials. a) Schematic diagram of the synthesis of ZnO/SiO_2_. b) TEM images of ZnO/SiO_2_. c,c_1_,c_2_) HRTEM image, EDX image, and lattice spacing diagram of ZnO/SiO_2_. d,e) XRD spectra and room temperature ESR spectra of SiO_2_, ZnO, ZnO/SiO_2_. f) XPS spectra (O 1s binding energy region) of ZnO, ZnO/SiO_2_.

A zinc oxide‐decorated silica catalyst precursor was synthesized using zinc acetate dihydrate and nanosized silica (30 nm) as starting materials, with L‐ascorbic acid serving as a structure‐directing agent in an ethanol–water solvent system. Upon pyrolysis, the precursor was converted into the ZnO/SiO_2_ catalyst (**Figure** **1**a). Detailed experimental procedures are provided in the Methods section. Transmission electron microscopy (TEM) and scanning electron microscope (SEM) images revealed a uniform dispersion of ZnO nanoparticles on the SiO_2_ surface (Figures 1b; S1a, Supporting Information), which is attributed to the isotropic crystal growth of ZnO directed by _L_‐ascorbic acid.^[^ ^35^ ^]^ In contrast, unsupported ZnO formed nanosheet‐like structures with an average lateral size of ≈10–30 nm (Figures S1b and S2a, Supporting Information). High‐resolution TEM (HRTEM) images further confirmed the presence of defect‐rich regions in ZnO/SiO_2_ (Figure 1c,c_2_), indicative of coordinatively unsaturated Zn atoms.^[^ ^36^ ^]^ Energy‐dispersive X‐ray spectroscopy (EDX) mapping demonstrated the homogeneous distribution of Zn and Si elements (Figure 1c_1_), whereas pristine ZnO showed negligible defect features (Figure S2b,b_1_,b_2_, Supporting Information). Inductively coupled plasma (ICP) analysis indicates that the zinc doping content of the ZnO/SiO_2_ catalyst is 22%.

X‐ray diffraction (XRD) pattern confirmed the coexistence of hexagonal ZnO (PDF#36‐1451) and amorphous silica (PDF#29‐0085) phases in ZnO/SiO_2_ (Figure 1d). Electron paramagnetic resonance (EPR) spectra further verified the existence of Zn defects and oxygen vacancies in ZnO/SiO_2_, as evidenced by signals at g = 1.950 and g = 2.001 (Figure 1e).^[^ ^37^, ^38^ ^]^ For comparison, pristine ZnO only exhibited a weak signal corresponding to oxygen vacancies (g = 2.002). X‐ray photoelectron spectroscopy (XPS) analysis showed that the binding energy of Zn 2p_3/2_ in Zn─O─Si bonds (1021.7 eV) was lower than that in Zn─O─Zn bonds (1021.3 eV), suggesting an altered electronic environment (Figure S3, Supporting Information).^[^ ^39^ ^]^ Moreover, the O 1s spectrum of ZnO/SiO_2_ exhibited a noticeable shift in binding energy from 530.0 and 531.5 eV in ZnO to 530.8 and 532.7 eV (Figure 1f), indicating that the formation of Zn─O─Si coordination bonds reduces the electron density around oxygen atoms.^[^ ^40^ ^]^ Collectively, these results demonstrate that ZnO/SiO_2_ is a structurally well‐defined catalyst featuring a high density of unsaturated Zn sites and oxygen vacancies.

### 2.2 Polyester Depolymerization Catalyzed by ZnO/SiO_2_

**Figure 2:**
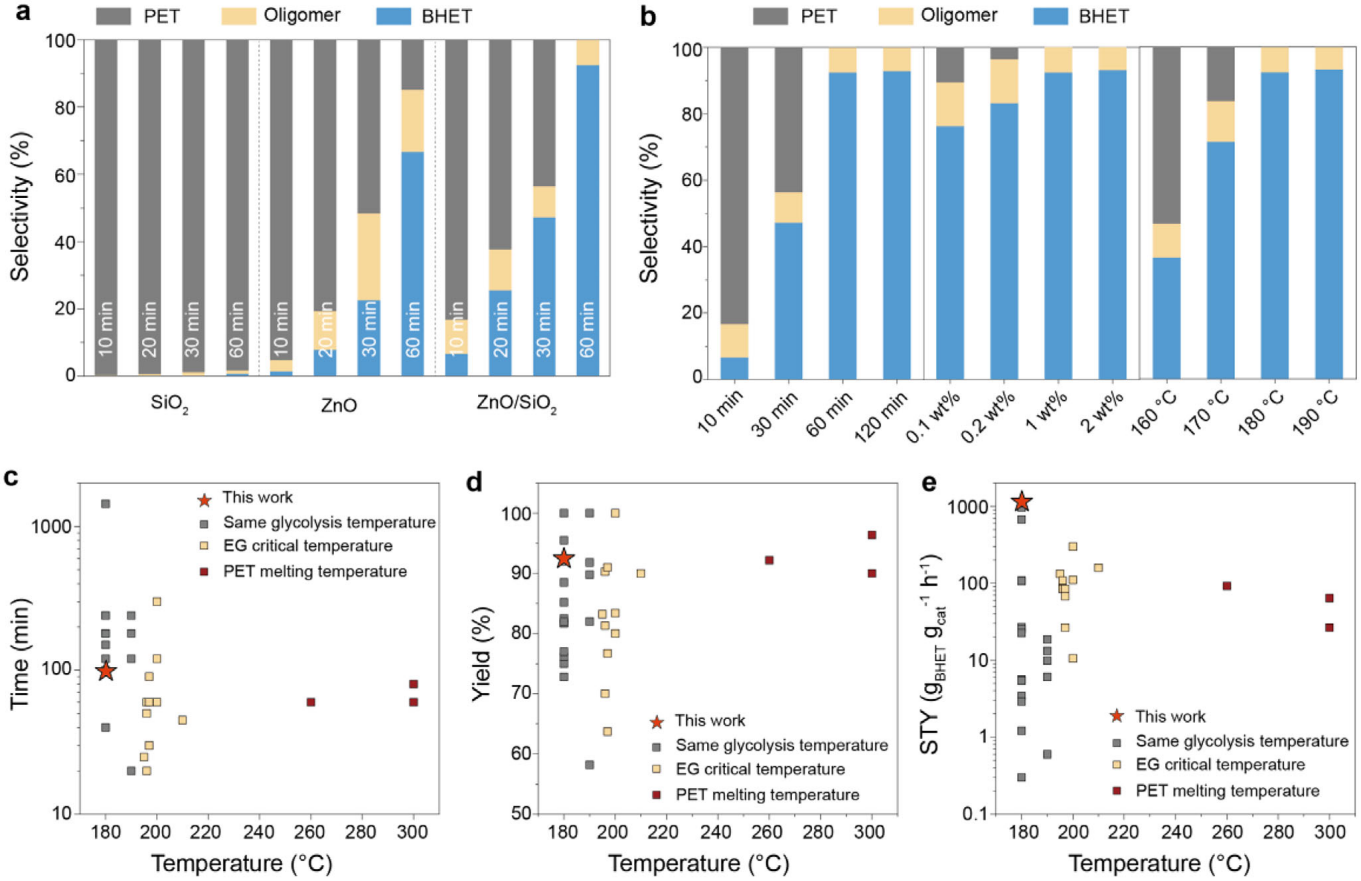
Depolymerization of PET. a) Catalytic performance of PET glycolysis over SiO_2_, ZnO, and ZnO/SiO_2_. Conditions: PET waste (1.0 g), EG (4 g), 180 °C, catalyst (10 mg). b) Optimizing glycolysis performance of PET over ZnO/SiO_2_. Conditions: PET waste (1.0 g), ZnO/SiO_2_ (1, 2, 10, and 20 mg), EG (4 g), temperature (160, 170, 180, and 190 °C), time (10 min, 30 min, 1 h, and 2 h). c–e) Comparison of the ZnO/SiO_2_ catalyst with other reported catalysts in terms of temperature‐time, temperature‐BHET yield, and temperature‐STY. The BHET yield was detected by liquid chromatography.

To evaluate the glycolysis performance of the catalysts, polyethylene terephthalate (PET, M_n_ = 28 kDa) was used as the model substrate at 180 °C, using SiO_2_, ZnO, and ZnO/SiO_2_ as catalysts (**Figure** **2**a). After the reaction, the mixture was cooled to room temperature, and the products were analyzed to determine their composition and yield. High‐performance liquid chromatography (HPLC) was employed to quantify the yields (Figure S4, Supporting Information), and nuclear magnetic resonance (NMR) spectroscopy was used to analyze product structure and purity (Figures S5 and S6, Supporting Information). In the presence of SiO_2_ and ZnO, PET conversion after 1 h was <2% and 85%, respectively, with corresponding bis(2‐hydroxyethyl) terephthalate (BHET) yields of <1% and 71.7%. In contrast, ZnO/SiO_2_ exhibited outstanding catalytic activity, achieving 100% PET conversion and 92.5% BHET yield (Figure 2a; Table S1, Supporting Information). Moreover, the increasing selectivity of BHET in the product mixture suggested that ZnO/SiO_2_ facilitates ester bond cleavage more effectively.

To comprehensively study the influencing factors of the polyester depolymerization process, the reaction conditions were systematically optimized. During the initial 30 min, the reaction primarily occurred in the amorphous regions of PET, forming nucleophilic oligomers and monomers that cooperatively accelerated the overall depolymerization. As a result, BHET yield rapidly increased to 92.5% (Figure 2b; Table S2, Supporting Information). As the reaction time was extended, the BHET yield exhibited minimal variation, indicating that the reaction had approached thermodynamic equilibrium. Remarkably, even at a low ZnO/SiO_2_ loading (0.1 wt.%), the PET conversion reached 89.5%, with a BHET yield of 78.3% (Figure 2b; Table S2, Supporting Information), indicating a high utilization efficiency of the ZnO defect sites on the SiO_2_ surface. These defect‐rich sites facilitate the effective adsorption and activation of both ethylene glycol and polyester chains, thereby promoting rapid polyester depolymerization. When the glycolysis reaction temperature exceeds the critical boiling point of ethylene glycol (197 °C), the hydroxyl groups of ethylene glycol are more readily activated by the catalyst to generate nucleophilic species, thereby facilitating the cleavage of ester bonds in PET. Remarkably, the ZnO/SiO_2_ catalytic system exhibited optimal activity at 180 °C (Figure 2b; Table S2, Supporting Information), indicating its ability to effectively adsorb and activate reactants even below the boiling point of ethylene glycol. Gel permeation chromatography (GPC) analysis revealed that the oligomers produced under the optimized reaction conditions were predominantly dimers, with a number‐average molecular weight (M_n_) of ≈450 *Da* (Figure S7, Supporting Information).

Compared with previously reported heterogeneous catalysts, the superior catalytic activity and selectivity of ZnO/SiO_2_ are more clearly demonstrated through systematic comparisons of time–temperature, BHET yield–temperature relationships, and space–time yield (STY)–temperature (Figure 2c–e; Table S3, Supporting Information). Condition optimization experiments confirm that temperature and reaction time are the key parameters governing the efficiency of polyester glycolysis. Notably, ZnO/SiO_2_ enables efficient depolymerization under milder conditions compared to other heterogeneous systems (Figure 2c). The ZnO/SiO_2_ catalyst achieves a significantly higher BHET yield at 180 °C, underscoring its high activity at relatively low temperatures (Figure 2d). The space–time yield (STY), defined as the mass of product generated per unit time. For a comprehensive performance comparison with previous studies conducted under varied reaction conditions, glycolysis results were classified into three categories: 1) equivalent glycolysis temperature (180, 190 °C); 2) the boiling point of ethylene glycol; and 3) the melting point of PET (Figure 2e). ZnO/SiO_2_ demonstrates catalytic activity at least one order of magnitude higher than most reported systems, underscoring its substantial advantage in polyester recycling.

### 2.3 Mechanism of Polyester Depolymerization

**Figure 3:**
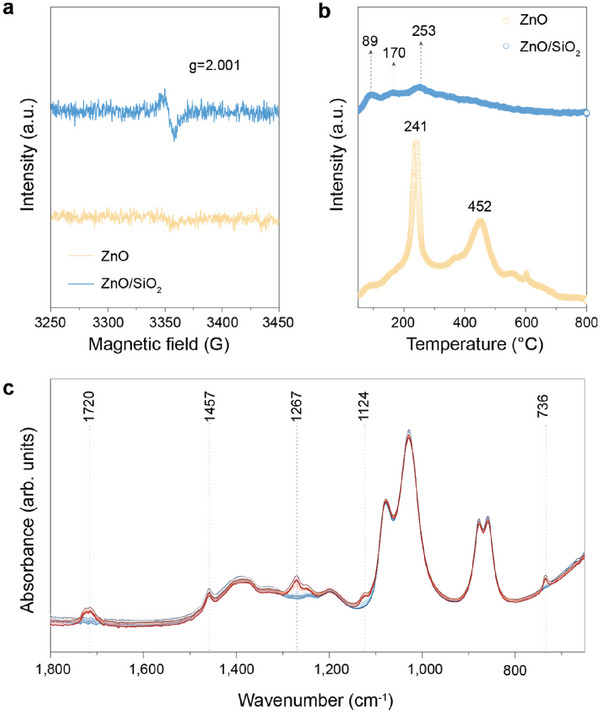
Mechanism of PET glycolysis over ZnO/SiO_2_. a) Low‐temperature EPR spectra of commercial ZnO and ZnO/SiO_2_ at 100 K. b) O_2_‐TPD profiles of commercial ZnO and ZnO/SiO_2_. c) In situ high‐temperature infrared spectroscopy to determine the PET glycolysis in HOCH_2_CH_2_OH under an air atmosphere.

To elucidate the nature of the active sites on ZnO and ZnO/SiO_2_ catalysts, low‐temperature electron paramagnetic resonance (EPR) spectroscopy was performed at 110 K to probe surface‐adsorbed oxygen species. A pronounced signal at g = 2.001 was observed for ZnO/SiO_2_, attributable to O_3_^−^ species (**Figure** **3**a), whereas no such signal appeared for ZnO.^[^ ^41^ ^]^ Upon heating to 150 K, O_3_^−^ species are known to dissociate into atomic oxygen (O^−^), suggesting that O^−^ is the dominant active oxygen species in the ZnO/SiO_2_ system under reaction conditions. This inference was corroborated by O_2_ temperature‐programmed desorption (O_2_‐TPD) analysis (Figure 3b). In the O_2_‐TPD spectra, desorption peaks below 100 °C, between 100–300 °C, 300–400 °C, 400–550 °C, and above 550 °C correspond to physiosorbed O_2_ (O_2(ad)_), superoxide (O_2_^−^), atomic oxygen (O^−^), and lattice oxygen (O_latt_^2−^), respectively.^[^ ^42^, ^43^ ^]^ Notably, ZnO/SiO_2_ exhibited a weak O_2_^−^ peak at 253 °C and a strong O^−^ peak at 440 °C, indicating that O^−^ is the predominant and most stable reactive oxygen species, which is consistent with the EPR results. We attribute these oxygen species to adsorption at the oxygen vacancy formed on the ZnO/SiO_2_ surface.

To investigate the interactions of ZnO/SiO_2_ with ethylene glycol (HOCH_2_CH_2_OH) and PET, in situ Fourier‐transform infrared (FT‐IR) spectroscopy was employed to monitor the evolution of functional groups during glycolysis (Figure 3c). During the reaction, increases in the absorption bands at 1032 cm^−1^ (ν(C─OH)) and 1457 cm^−1^ (ν(C─O─H)) were observed, indicating the formation of a nucleophilic intermediate. Concurrently, new bands emerged at 1720 cm^−1^ (ν(C═O)), 1267 cm^−1^ (ν(C─O)), and 1124 cm^−1^ (ν(C─OH)), signifying the cleavage of PET ester bonds and the formation of BHET.^[^ ^44^, ^45^ ^]^ This suggests that the C─O bonds of PET were attacked by the nucleophilic intermediate, initiating depolymerization.

Based on these results, we propose the following mechanism (Figure S8, Supporting Information): HOCH_2_CH_2_OH molecules are adsorbed at surface defect sites of ZnO/SiO_2_, where they form activated HOCH_2_CH_2_OH nucleophiles. The coordinatively unsaturated Zn^2+^ centers on the ZnO/SiO_2_surface activate the carbonyl oxygen of PET, inducing a partial positive charge on the carbonyl carbon. Nucleophilic attack by HOCH_2_CH_2_OH then leads to cleavage of the ester C─O bond and formation of BHET. Simultaneously, the liberated chain ends are stabilized by protons, forming additional HOCH_2_CH_2_OH. This synergistic activation and nucleophilic substitution pathway accounts for the high efficiency of PET depolymerization catalyzed by ZnO/SiO_2_.

### 2.4 Polyester Depolymerization Catalyzed by ZnO/SiO_2_

**Figure 4:**
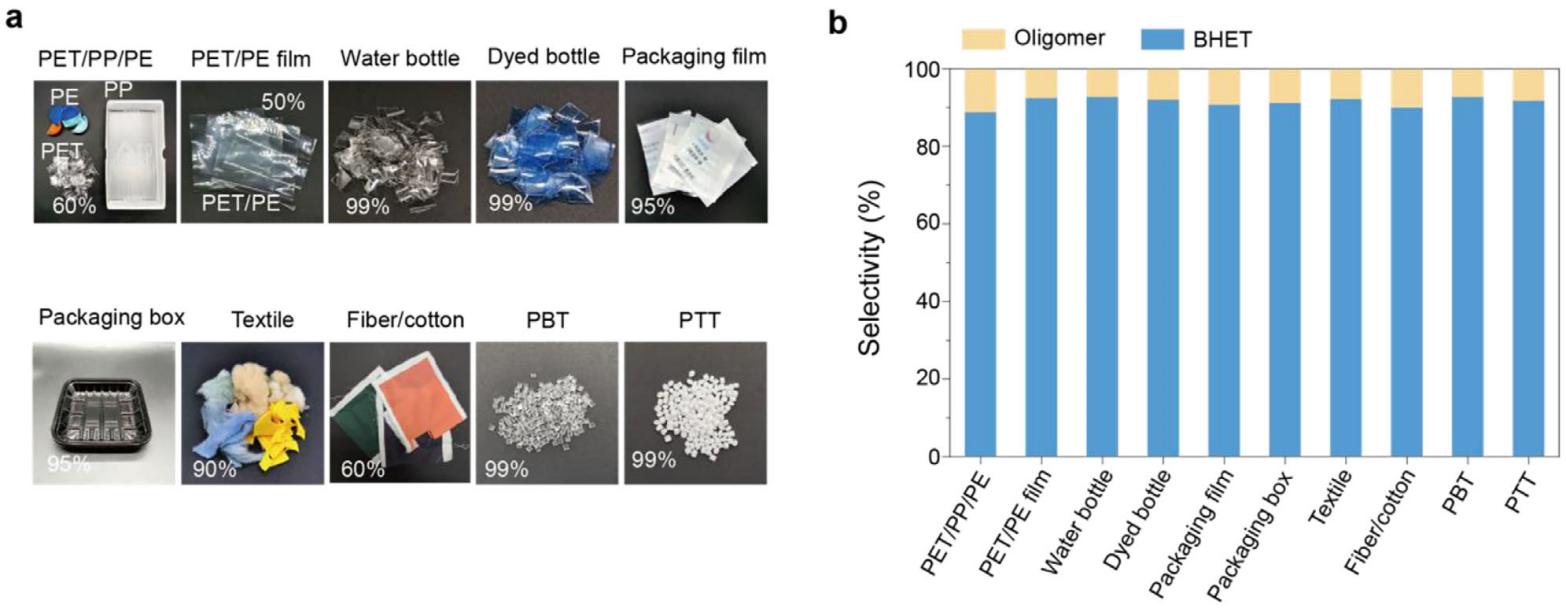
Glycolysis of real polyester waste over ZnO/SiO_2_. a,b) Photographs and yield of mixed polyester waste glycolysis. Conditions: polyester waste (PE/PP/PE, PET/PE film, water bottle, dyed bottle, packaging film, packaging box, textile, fiber/cotton, PBT, PTT, 1.0 g), ZnO/SiO_2_ (10 mg), EG (4 g), 180 °C, 1 h.

Efforts to recycle real‐world polyester waste are essential to mitigating global plastic pollution and facilitating the transition toward sustainable plastic management. The catalytic performance of the ZnO/SiO_2_ system was evaluated using a variety of representative post‐consumer polyester waste sources, including beverage bottles, discarded fibers, blended textiles, packaging films, and mixed polyester‐based plastics (**Figure** **4**a). Depolymerization of PET in the presence of common non‐polar plastics such as polyethylene (PE) and polypropylene (PP) revealed that ZnO/SiO_2_ maintained high catalytic efficiency, achieving complete PET depolymerization with BHET yields exceeding 90%, indicating negligible interference from PE or PP components (Figure 4b; Table S4, Supporting Information). Similarly, 100% depolymerization and >90% BHET yields were obtained for a wide range of real wastes, including clear and colored bottles, packaging materials, and textile waste, confirming that typical post‐consumer impurities and additives do not adversely affect the catalyst performance (Figure 4b; Table S4, Supporting Information). One notable challenge observed was the presence of persistent dye residues in the product stream, which can be effectively removed via activated carbon adsorption. Furthermore, structurally similar polyesters such as polybutylene terephthalate (PBT) and polytrimethylene terephthalate (PTT) were also efficiently depolymerized, with BHET yields surpassing 90% (Figure 4b; Table S4, Supporting Information). These results demonstrate the broad applicability and robustness of the ZnO/SiO_2_ catalyst in the depolymerization of diverse real‐world polyester wastes, underscoring its potential in practical recycling applications. Importantly, ZnO/SiO_2_ also demonstrated excellent performance in large‐scale depolymerization of PET flakes (50 g), yielding over 60 g of recovered BHET (Table S4, Supporting Information).

The ZnO/SiO_2_ catalyst enabled efficient depolymerization and recycling of a wide range of real‐world polyester plastics, a comprehensive assessment of its long‐term stability is essential for practical application. After five consecutive catalytic runs using PET as the substrate, a slight decline in BHET yield was observed (Figure S9, Supporting Information). Notably, complete PET conversion could still be achieved by extending the reaction time to 2 h, affording BHET yields above 90 %. Furthermore, the catalytic activity could be fully restored to its initial level through high‐temperature calcination. The regeneration procedure is detailed in the Supporting Information. XRD patterns of the regenerated ZnO/SiO_2_ catalyst showed no additional peaks or peak shifts (Figure S10, Supporting Information), indicating that the catalyst retained its structural integrity during the regeneration cycles.

### 2.5 Life Cycle Assessment (LCA) and Techno‐Economic Assessment (TEA)

A cut‐off approach was adopted to assess the environmental, social, and economic impacts of the waste polyester recycling process.^[^ ^46^, ^47^, ^48^ ^]^ Activity data were primarily derived from a conceptual plant model constructed using Aspen software (Figure S11 and Table S5, Supporting Information), with an annual processing capacity set at 100 000 tons. The polyester recycling process was modeled in two stages: 1) collection, transportation, and pretreatment of polyester waste; and 2) depolymerization, encompassing catalyst synthesis, polyester depolymerization, and product separation. To enhance model fidelity, data from experiments, literature reports, and industrial operations were integrated, while background inventory data (e.g., energy and chemical production) were sourced from the ecoinvent v3.7 database (Tables S6 and S7, Supporting Information).

**Figure 5:**
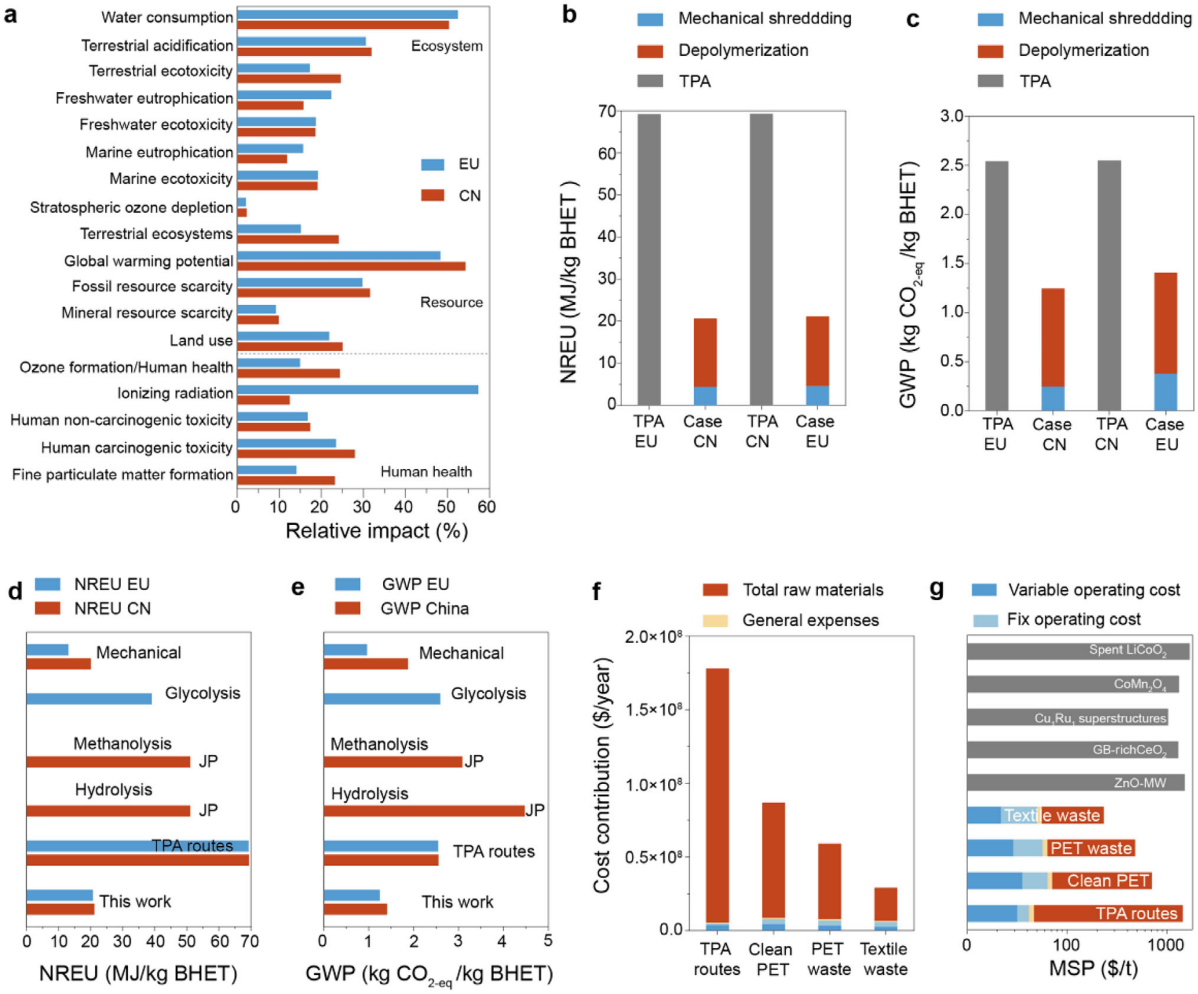
Life‐cycle assessment (LCA) and techno‐economic analysis (TEA) of PET glycolysis. a) Comparative life‐cycle assessment results of the case‐CN or case‐EU recycling route and TPA‐CN or TPA‐EU route. Comparison of b) non‐renewable energy use (NREU) and c) global warming potential (GWP). d,e) Comparison of NREU and GWP in different routes for recycling PET. EU: Europe, CN: China, JP: Japan. f) Analysis of cost contribution based on TPA routes, clean PET, waste PET, and clean textiles recycling processes. g) Comparison of minimum selling prices based on TPA routes, clean PET, waste PET, clean textiles, and references.

Using BHET recovery as the functional unit, the environmental performance of the polyester recycling process was benchmarked against that of the TPA synthesis route in terms of human health, resource, and ecosystem (Figure S12, Supporting Information). Owing to efficient energy integration and high recovery rates, the recycling pathway resulted in substantial reductions across 18 environmental impact categories (**Figure** **5**a; Tables S8–S11, Supporting Information). Particularly noteworthy are the global warming potential (GWP) and non‐renewable energy use (NREU). In Europe, the GWP was 1.242 kg CO_2‐eq_ kg^−1^, ≈104% lower than that of the TPA synthesis route, while NREU was 20.617 MJ kg^−1^, representing a reduction of ≈235% (Figure 5b,c; Tables S12–S21, Supporting Information). In China, the GWP was 1.403 kg CO_2‐eq_ kg^−1^, ≈81% lower than that of the TPA route, while NREU was 21.089 MJ kg^−1^, representing a reduction of ≈228% (Figure 5b,c; Tables S12–S21, Supporting Information). Energy and chemical inputs were identified as the dominant contributors to the total environmental burden. Accordingly, strategies such as substituting electric heating with steam, optimizing heat exchanger design to recover waste heat, and using recycled over virgin feedstocks are critical for minimizing environmental impacts. Furthermore, comparison with other recycling strategies (i.e., methanolysis, hydrolysis, and glycolysis) highlight the superior GWP and NREU profiles of our process (Figure 5d,e). Apart from approaching the performance of mechanical recycling, our route outperformed chemical alternatives across metrics.

A techno‐economic analysis was conducted to compare the total capital investment and unit production cost of the polyester recycling process with those of the conventional TPA synthesis route (Figure 5f; Tables S22–S29, Supporting Information). The recycling process entails additional large‐scale infrastructure, including depolymerization reactors and flash tanks for solvent separation, thereby increasing both direct and indirect capital expenditures. By simulating multiple scenarios, we identified the principal factors influencing the minimum selling price (MSP) of recycled BHET. Among various uncertainties, raw material cost becomes the most critical determinant of MSP. Notably, the unit production cost of BHET decreased from 700.5 $ t^−1^ (clean PET) to 233.6 $ t^−1^ (textile waste), corresponding to a ≈2 times reduction (Figure 5g; Tables S30–S34, Supporting Information). This value is significantly lower than the average market price of virgin BHET (1435.3 $ t^−1^), thus enhancing the economic feasibility of the recycling process. Moreover, the cost of BHET production via this route is substantially lower than that reported for other polyester glycolysis recycling strategies (Figure 5g). Taken together, the polyester recycling process not only offers environmental, ecological, and health‐related advantages over the TPA route, but also demonstrates superior economic performance.^[^ ^31^, ^32^, ^48^, ^49^, ^50^ ^]^ These results underscore the potential of this approach as a sustainable, cost‐effective solution for large‐scale polyester waste valorization.

## 3 Conclusion

In summary, we have developed a silica‐mediated strategy for synthesizing defect‐rich ZnO nanomaterials, which serve as efficient catalysts for the chemical recycling of polyester waste plastics. The ZnO/SiO_2_ catalyst exhibits excellent catalytic activity (STY = 1009.3 g_BHET_ g_cat_^−1^ h^−1^) and recyclability over five cycles. The high density of Zn^2+^ defect sites and oxygen vacancies facilitates the activation of both ethylene glycol and polyester, leading to the formation of nucleophilic species. These species subsequently attack the ester bonds of the polyester backbone, promoting C─O bond cleavage and yielding BHET. LCA demonstrates that this method significantly reduces carbon emissions and the consumption of non‐renewable resources, while also minimizing impacts on human health, ecosystems, and resource depletion compared to conventional TPA production. TEA indicates that this process is suitable for converting various qualities of polyester waste into recycled BHET. This work highlights the potential of defect‐engineered heterogeneous catalysts for the sustainable and economically viable recycling of polyester waste, offering a promising route toward improved plastic life cycle management.

## Conflict of Interest

The authors declare no conflict of interest.

## Supporting information



Supporting Information

## Data Availability

The data that support the findings of this study are available in the supplementary material of this article.
