# Supplementary material for: Catalytic Depolymerization of Polyester Waste via Zinc Oxide‐Decorated Silica
Source: Adv Sci (Weinh). 2025 Oct 27;13(3):e14922. doi: 10.1002/advs.202514922 (PMC12806222; doi:10.1002/advs.202514922)
Supplement: Supplementary file 1 — Supporting Information [file ADVS-13-e14922-s001.docx]

Supporting Information

Catalytic Depolymerization of Polyester Waste via Zinc Oxide-Decorated Silica

Jingjing Cao^1*^, Huaxing Liang^2^, Wei Chen^1^, Jiachen Dong^1^, Zhiguang Li^1^, Shaohai Fu^1,3^

Table of Contents

Supporting experimental methods

Supporting figures

Figure S1. (a, b) Scanning electron microscope image (SEM) of ZnO/SiO_2_ and ZnO.

Figure S2. (a) TEM images of ZnO. (b, b_1_, b_2_) HRTEM image, EDX image, and lattice spacing diagram of ZnO.

Figure S3. XPS spectra (Zn 2p binding energy region) of ZnO, ZnO/SiO_2_.

Figure S4. HPLC patterns of the product.

Figure S5. 1H NMR spectrum of a product recorded in DMSO-*d_6_*.

Figure S6. 13C NMR spectrum of a product recorded in DMSO- *d_6_*.

Figure S7. GPC analysis results of dimers.

Figure S8. Schematic illustration of PET glycolysis catalyzed by ZnO/SiO_2_ catalyst.

Figure S9. Catalyst stability. Conditions: PET waste (1.0 g), ZnO/SiO_2_ (10 mg) or *r*-catalyst, EG (4 g), 180°C, 1 h.

Figure S10. XRD pattern of the ZnO/SiO_2_ and regenerated ZnO/SiO_2_ (*r*-ZnO/SiO_2_).

Figure S11. Flowsheet diagram from Aspen model for PET glycolysis Case 1.

Figure S12. Flowsheet diagram from Aspen model for virgin BHET route Case 2.

Supporting tables

Table S1. Selectivity of products from catalytic glycolysis of PET in different reaction conditions.

Table S2. Optimization of catalytic glycolysis of PET.

Table S3. Comparison of reported catalysts for PET glycolysis.

Table S4. Catalytic performance of different polyester plastics and mixed plastics.

Table S5. Goal and scope of LCA.

Table S6. NREU values of each raw material of PET glycolysis used in the Open LCA.

Table S7. GWP values of each raw material of PET glycolysis used in the Open LCA.

Table S8. LCA results of case-CN recycling route.

Table S9. LCA results of case-EU recycling route.

Table S10. Comparative LCA results of case-CN recycling route and virgin BHET route.

Table S11. Comparative LCA results of case-EU recycling route and virgin BHET route.

Table S12. LCA results in glycolysis of post-consumer PET bottles, functional unit = 1 kg recycled BHET.

Table S13. LCA results in TPA route to produce BHET (virgin BHET route), functional unit = 1 kg virgin BHET.

Table S14. Material and energy input-output of PET bottles glycolysis, functional unit = 1 kg BHET flakes.

Table S15. Mass balance of post-consumer PET bottles glycolysis.

Table S16. Mass balance of virgin BHET route.

Table S17. Details of the utilities with post-consumer PET bottles glycolysis and virgin BHET route.

Table S18. Utility of post-consumer PET bottles glycolysis.

Table S19. Utility of virgin BHET route.

Table S20. Energy balance of post-consumer PET bottles glycolysis.

Table S21. Energy balance of virgin BHET route.

Table S22. Total equipment cost of PET recycling.

Table S23. Total equipment cost of virgin BHET-CN route.

Table S24. Total direct cost, total indirect cost, and total capital investment cost of clean PET, PET wastes, and textile waste recycling.

Table S25. Variable operating cost, fix operating cost, general expenses and total raw materials of clean PET, PET wastes, and textile waste recycling.

Table S26. Total direct cost, total indirect cost, and total capital investment cost of TPA route to produce BHET.

Table S27. Variable operating cost, fix operating cost, general expenses of TPA route to produce BHET.

Table S28. Total direct cost, total indirect cost, land, working capital and total capital investment cost of PET waste recycling.

Table S29. Total direct cost, total indirect cost, land, working capital and total capital investment cost of TPA route to produce BHET.

Table S30. Utility operation cost of clean PET recycling.

Table S31. Utility operation cost of PET waste recycling.

Table S32. Utility operation cost of textile waste recycling.

Table S33. Utility operation cost of TPA route to produce BHET.

Table S34. MSP of PET recycling, TPA route to produce BHET, and literature.

Supporting references

Experimental methods

Chemical reagents and materials

Plastics: PET, PBT, and PTT were purchased from Taobao Mall. PET wastes were obtained from various PET products, including transparent (PET accounts for 99%) and colored post-consumer PET bottle (PET accounts for 99%), packaging boxes (PET accounts for 95%) and film (PET accounts for 95%), textile (PET accounts for 90%), and fiber/cotton (PET accounts for 60%). Other plastic waste is collected from landfills, including PE, PP.

Reagents: Zinc acetate dihydrate, 30 nm silica (SiO_2_), ethanol, ethylene glycol, and _L_-ascorbic acid were purchased from Shanghai Macklin Biochemical Co., Ltd. Deionized water was purified with a Milli-Q system (Laboratory-made). All chemical reagents were used without further purification.

Materials synthesis

**Synthesis of zinc oxide (ZnO).** Zn(OAc)_2_·2H_2_O (10 mmol) solution was mixed with 100 mL deionized water. Next, 0.02 M NaOH (200 mL) solution was drop adding to form white flocculent precipitates under stirring for 1 h. The product was then harvested by centrifugation, washed several times with ethanol and water, and dried in a vacuum oven.

**Synthesis of zinc oxide/silica (ZnO/SiO_2_).** Zn(OAc)_2_·2H_2_O (10 mmol) and SiO_2_ (1 g, ~30 nm) were dispersed in a mixed solvent of deionized water and ethanol (H2O: EtOH = 1:2, 100 mL), and the resulting mixture was transferred to a three-necked flask and stirred at 80 °C for 30 min. Subsequently, a 0.02 M NaOH aqueous solution (200 mL) was added dropwise to adjust the pH to approximately 10, and stirring was continued for an additional 30 min. L-ascorbic acid (10 mmol) was then added, and the reaction was maintained at 80 °C for 12 h. Upon completion, the precipitate was collected by filtration, washed three times with deionized water, and dried at 60 °C overnight. The resulting powder was calcined in air at 350 °C for 2 h with a heating rate of 5 °C min–1. The final product was denoted as ZnO/SiO_2_.

**Glycolysis of PET:** 1.0 g of PET, 4 g of ethylene glycol, and the appropriate catalyst were added to a 10 ml single-necked flask under a stirring speed of 300 rpm. The mass fraction of PET and catalyst was 0.1wt%–2wt%. Then, the reactor was heated to 180 °C for 1 h. After the reaction, filter to remove the catalyst and unreacted PET. Deionized water was added to the filtrate, and the filtrate was collected after rapid stirring and filtration. The PET conversion rate and BHET yield were calculated according to formula (1). The BHET yield was analyzed by HPLC. The space-time yield (STY) was calculated according to formula (3). Selectivity was calculated according to formula (4).

$\mathrm{Conversion}_{\mathrm{PET}}=\frac{m(PET input)-m(PET residue)}{m(PET input)}\times100\%$ (1)

$\mathrm{Yield}_{BHET}=\frac{n \left( \mathrm{BHET} \right)}{theoretical n \left( \mathrm{BHET} \right)}\times100\%$ (2)

$\mathrm{STY}=\frac{m(BHET)}{m(cat)\times t}$ (3)

$\mathrm{Selectivity}_{BHET}=\frac{n \left( \mathrm{BHET} \right)}{\sum n \left( \mathrm{BHET} \right)}\times100\%$ (4)

***In situ* attenuated total reflectance (ATR) infrared spectrometric experiments.** The operando IR spectra were recorded using a ReactIRTM 45 m spectrometer (Mettler Toledo) connected to a 30 mL reactor at 180 °C. Collect sample spectra to record the background, which serves as the baseline for subsequent measurements. Following this, after ethylene glycol was introduced into the reactor, the temperature was stepwise increased from room temperature to180 °C at a ramp rate of 20 °C min^–1^ and maintained at this temperature for 60 minutes. Typical signals indicative of intermediates formed during the depolymerization processes were diligently captured and analyzed using FTIR spectroscopy.

**Analysis of products.** BHET was analyzed quantitatively on a Waters measured with a C18 column (5 μm, 250 × 4.6 mm^2^, Agilent) and an ultraviolet detector at 254 nm. The mobile phase was methanol/water (v/v = 50/50) for a running time of 15 min at 30 °C, a flow rate of 0.5 mL min^−1^, and an injection volume of 10 μL.

**Characterization**

Scanning electron microscope (SEM) images were characterized by GeminiSEM 500 instrument using a at an accelerating voltage of 10 kV. Transmission electron microscope (TEM), High-resolution transmission electron microscopy (HRTEM) images and the corresponding energy dispersive X-ray spectroscopy (EDX) element mapping were acquired by a JEM-ARM200F instrument operated at 200 kV. XRD patterns were recorded on a Rigaku D/MAX-2500 diffractometer using a filtered Cu-Kα radiation source (λ = 1.54056 Å). The electron spin resonance (ESR) measurements were recorded on a JES-FA200 model spectrometer. The X-ray photoelectron spectroscopy (XPS) characterization was performed on the soft X-ray spectroscopy beamline in the Thermo Scientific ESCALAB 250Xi. The ZnO/SiO_2_ catalyst was analyzed using inductively coupled plasma optical emission spectroscopy (ICP-OES, Thermo Fisher iCAP RQ) to quantify the zinc ion (Zn2+) concentration. 1H-NMR and 13H-NMR spectra of BHET were collected by an AVI 400 MHz NMR spectrometer (Bruker). The O_2_-temperature-programmed desorption (TPD) measurement was determined on an automatic chemical adsorption instrument (Micromeritics ASAP 2020).

**Comprehensive Life-Cycle Assessment (LCA) of PET recycling.**

The recycling process was simulated using Aspen Plus V11 with the POLYSL model. We evaluated the impacts of the entire process on factors, such as human health, ecosystems, and resources using OpenLCA (Version, 1.10.3). We emphasize that the primary objective of the LCA was to compare the relative environmental impacts between conventional PET disposal methods and our proposed recycling process. While updates to the background database may influence the absolute values, the comparative trends and overarching conclusions are expected to remain valid. Our system boundary encompasses recycling framework, including collection and transportation and encompassing depolymerization. To gain a broader understanding of the environmental impacts in different global contexts, we evaluated the environmental impacts in two different geographical regions (China and Europe), with a specifically focusing on NREU and GWP. The chemical recycling processes were simulated on an industrial scale with an annual treatment of 100,000 tons of waste PET, using Aspen Plus V11 to obtain the mass balance and energy consumption. More details on the data and assumptions we applied in the LCA analysis are documented in the Supporting Note 2, 3 sections.

**Details of techno-economic analysis (TEA).**

The Aspen Process Economic Analyzer V11 was used to determine the capital and operating costs for conventional chemical plants. Minimum selling price (MSP), i.e., the selling price of the product when the net present value is zero. Heat integration for BHET monomer production was performed using Aspen Energy Analyzer (Aspen Technology 2019). Scenario analysis of key process parameters was performed to account for uncertainties. Additional TEA assumptions are provided in Supporting Note 4.

**
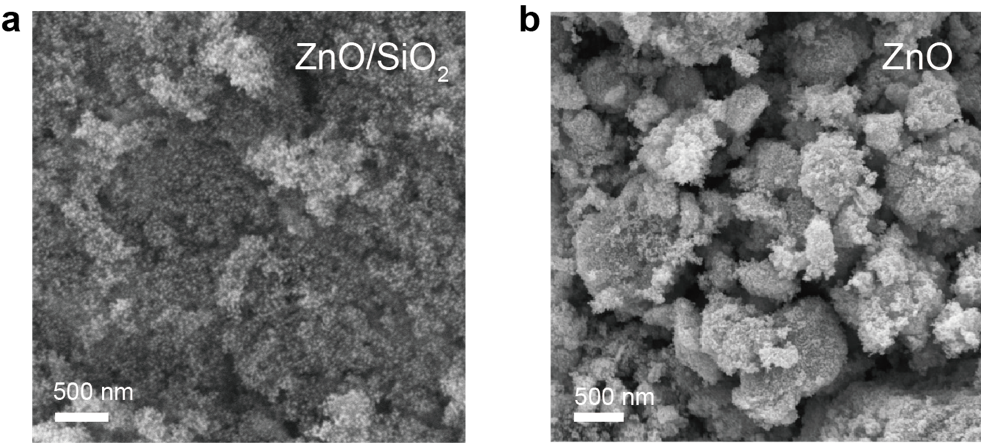
**

Figure S1. (a, b) Scanning electron microscope image (SEM) of ZnO/SiO_2_ and ZnO.


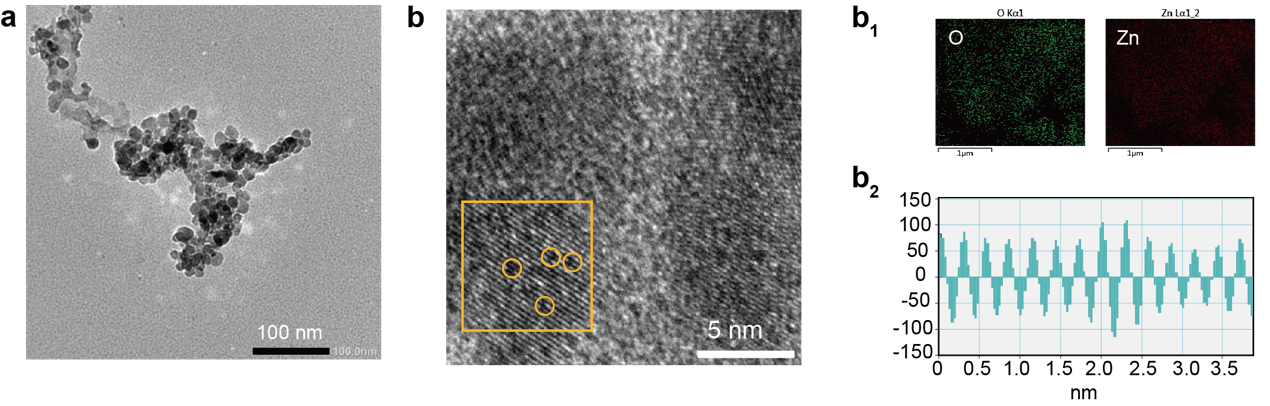


Figure S2. (a) TEM images of ZnO. (b, b_1_, b_2_) HRTEM image, EDX image, and lattice spacing diagram of ZnO.

**
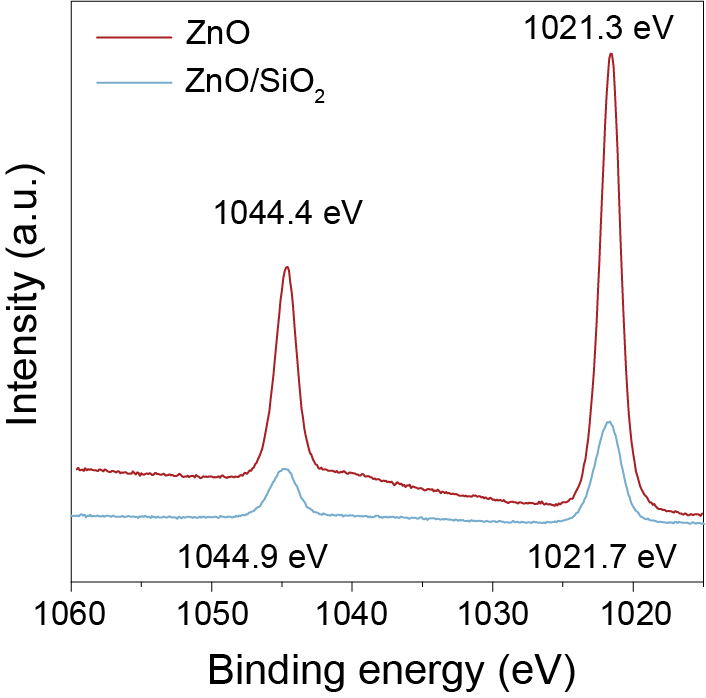
**

Figure S3. XPS spectra (Zn 2p binding energy region) of ZnO, ZnO/SiO_2_.


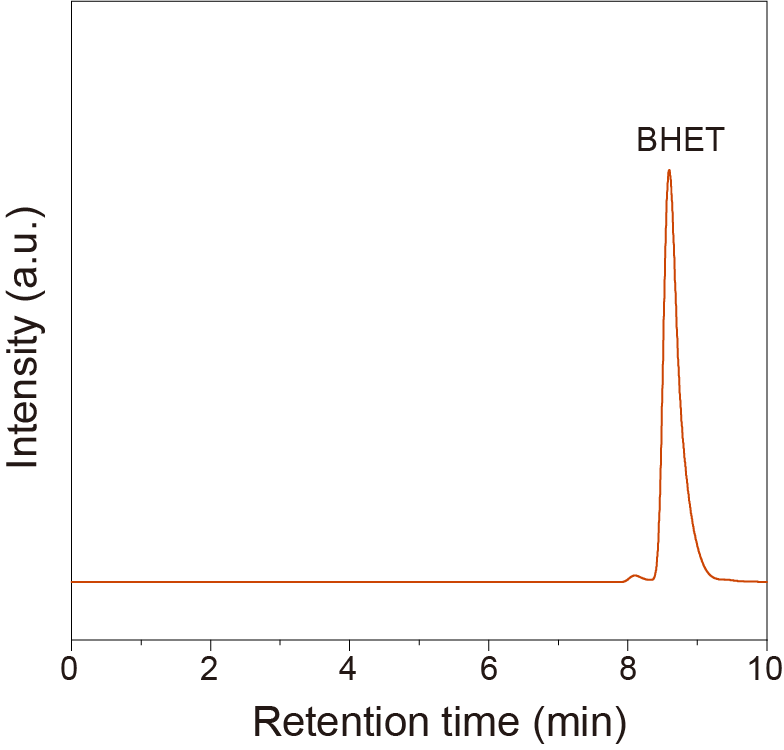


Figure S4. HPLC patterns of the product.


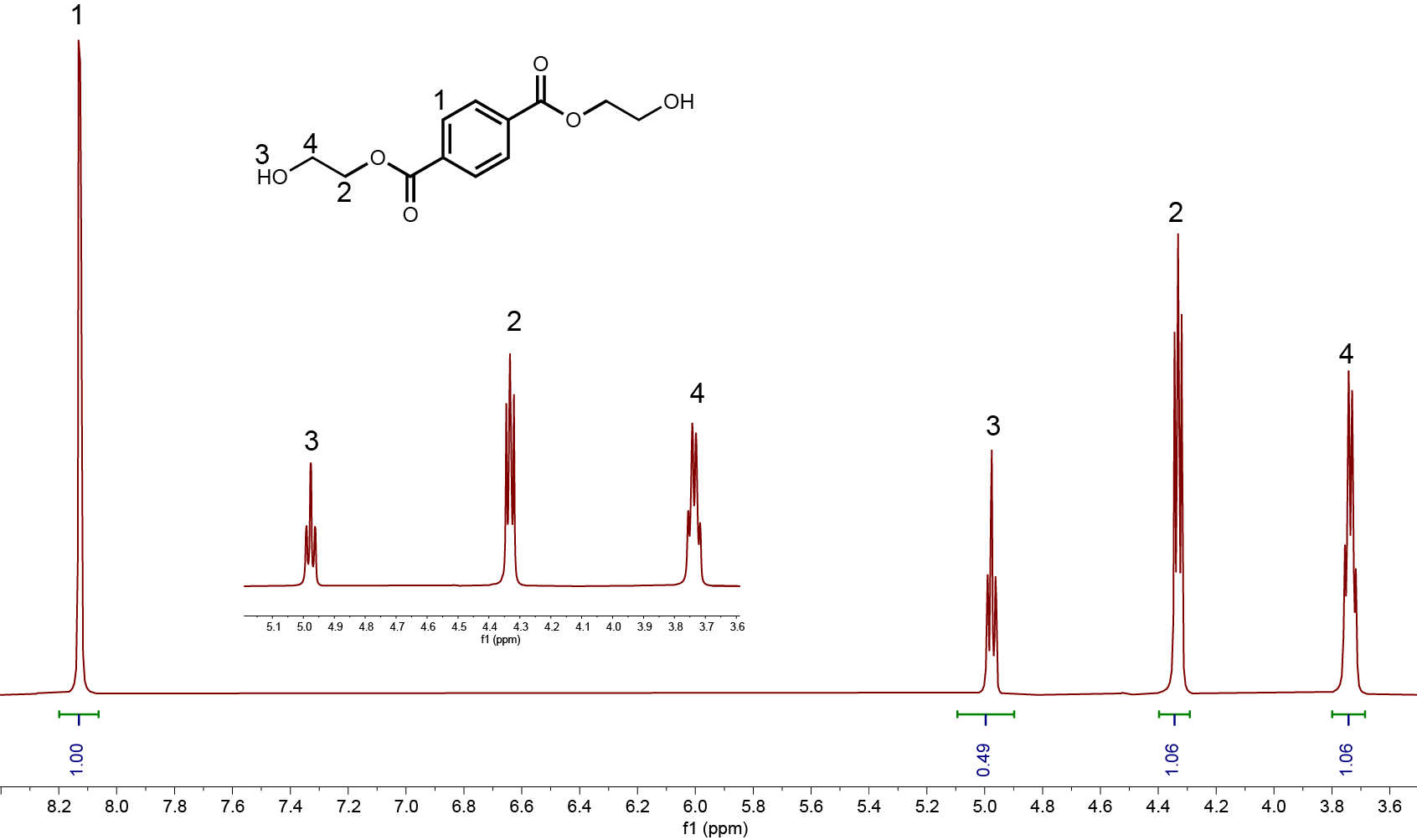


Figure S5. ^1^H NMR spectrum of a product recorded in DMSO-*d_6_*. ^1^H NMR (400 MHz, DMSO-*d_6_*, δ): 8.11 (s, 4H, CH), 4.99 (t, 2H; OH), 4.32 (t, 4H; O–CH_2_), 3.72 (q, 4H; CH_2_–OH)

The single signal observed at δ=8.11 ppm corresponds to the four symmetric aromatic protons of the benzene ring. Additionally, the triplet peak at δ=4.32 ppm and the quartet peak at δ=3.72 ppm represents the methylene protons of COO-CH_2_ and CH_2_-OH, respectively. The triplet peak at δ=4.99 ppm indicates the presence of protons from the hydroxyl group.


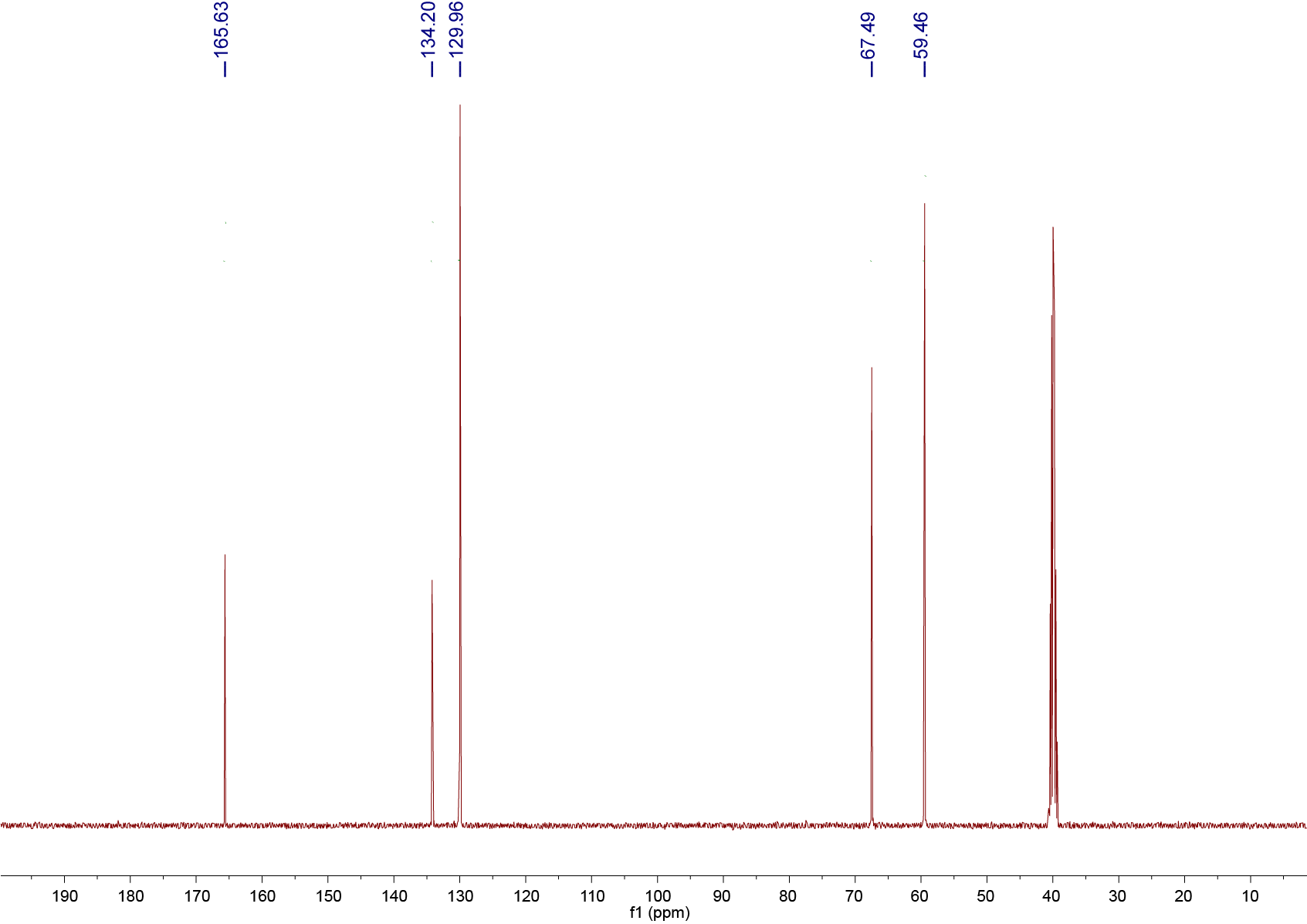


Figure S6. ^13^C NMR spectrum of a product recorded in DMSO-*d_6_*. ^13^C NMR (100 MHz, DMSO-d6, δ): δ 165.6, 134.2, 129.9, 67.4, 59.4 ppm.

In the ^13^C NMR spectrum, distinct signals appeared at δ 166.4 ppm (carbonyl carbon), δ 133.0 and 129.5 ppm (aromatic carbons), and δ 63.1 and 61.5 ppm (ethylene glycol carbons).


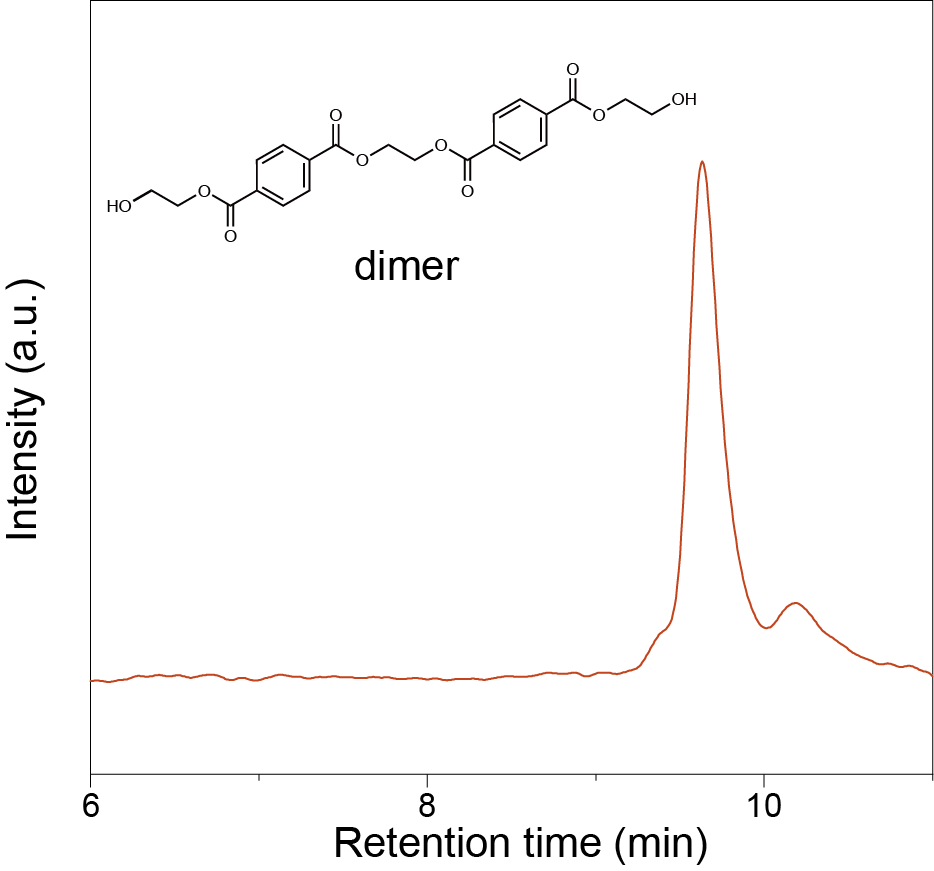


Figure S7. GPC analysis results of dimers.


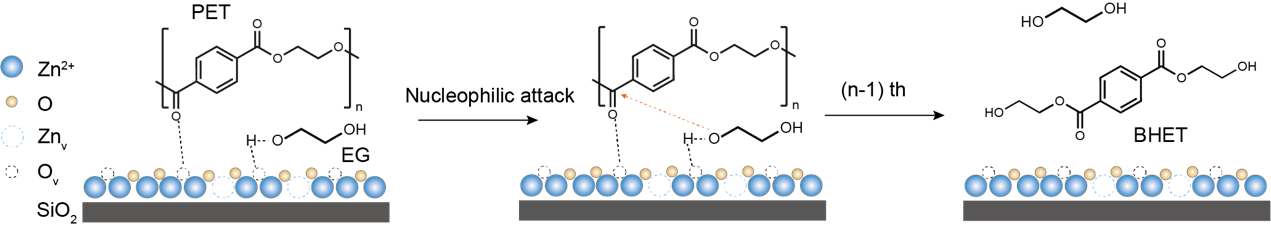


Figure S8. Schematic illustration of PET glycolysis catalyzed by ZnO/SiO_2_ catalyst.


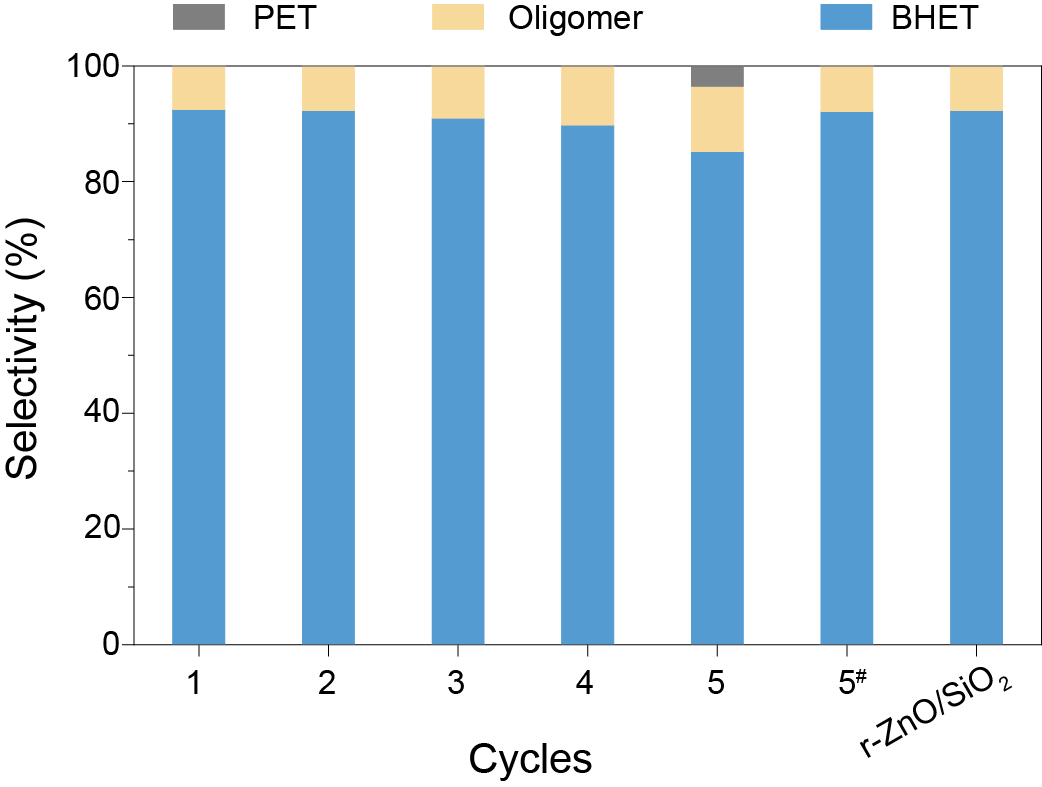


Figure S9. Catalyst stability. Conditions: PET waste (1.0 g), ZnO/SiO_2_ (10 mg) or *r*-catalyst, EG (4 g), 180°C, 1 h.


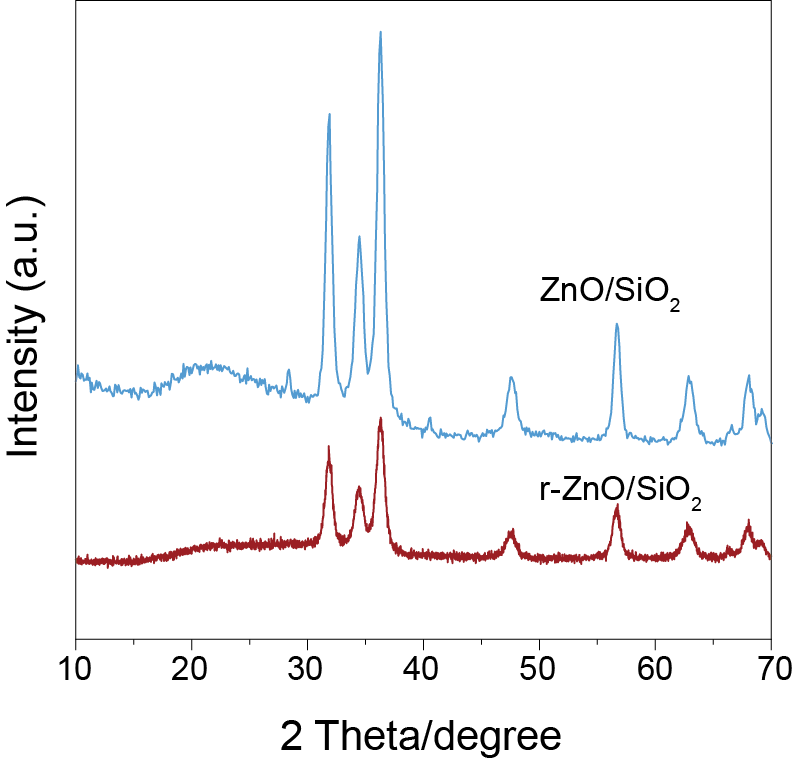


Figure S10. XRD pattern of the ZnO/SiO_2_ and regenerated ZnO/SiO_2_ (*r*-ZnO/SiO_2_).

Note 1: Catalyst regeneration is as follows: the reacted catalyst was stirred in ethylene glycol for 20 minutes at 300 rpm, then filtered to collect the catalyst. The catalyst was dispersed in ethanol, stirred for 1 hour, and then centrifuged to collect the product, followed by vacuum drying at 60 °C for 12 h. The resulting product was transferred to a tube furnace for calcination at 350 °C for 1 hour in an atmosphere of air, with a heating rate 2 °C/min.


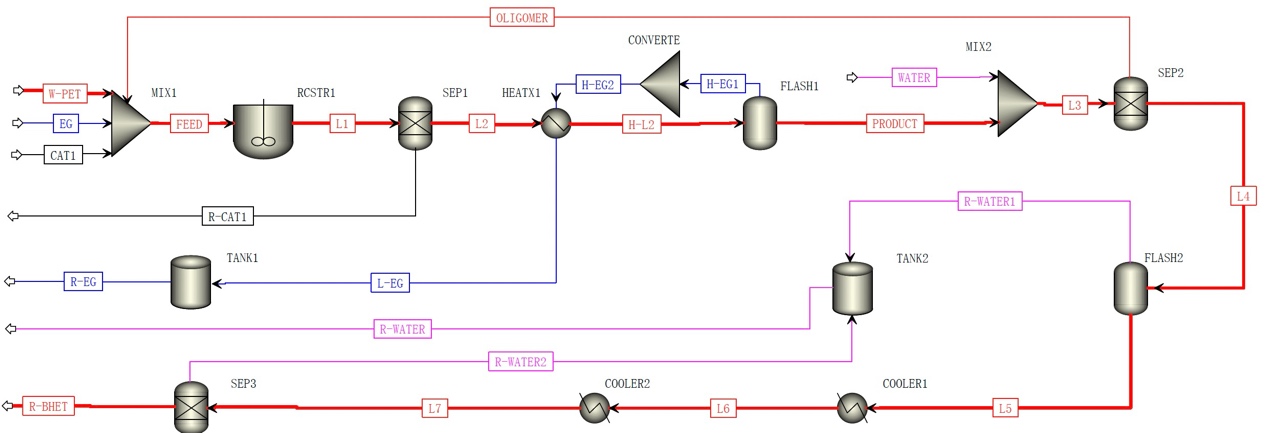


Figure S11. Flowsheet diagram from Aspen model for PET glycolysis Case 1.


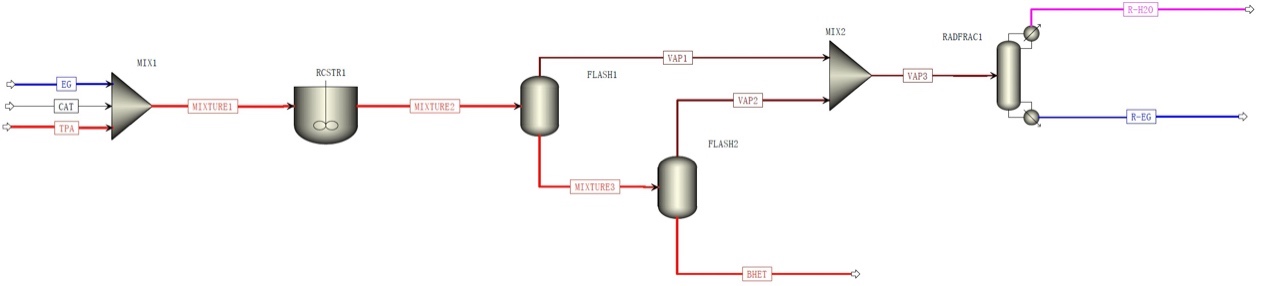


Figure S12. Flowsheet diagram from Aspen model for virgin BHET route Case 2.

Table S1. Selectivity of products from catalytic glycolysis of PET in different reaction conditions.

| Entry | Catalyst | t  (min) | PET  conv. (%) | BHET  yield (%) | Oligomer  yield (%) |
| --- | --- | --- | --- | --- | --- |
| 1 | SiO_2_ | 10 | 0.36 | 0.04 | 0.32 |
| 2 | SiO_2_ | 20 | 0.62 | 0.13 | 0.49 |
| 3 | SiO_2_ | 30 | 1.20 | 0.28 | 0.92 |
| 4 | SiO_2_ | 60 | 1.80 | 0.72 | 1.08 |
| 5 | ZnO | 10 | 4.80 | 1.50 | 3.30 |
| 6 | ZnO | 20 | 19.4 | 7.90 | 11.5 |
| 7 | ZnO | 30 | 48.5 | 22.6 | 25.9 |
| 8 | ZnO | 60 | 85.2 | 66.7 | 18.5 |
| 9 | ZnO/SiO_2_ | 10 | 16.8 | 6.70 | 10.1 |
| 10 | ZnO/SiO_2_ | 20 | 37.8 | 25.6 | 12.2 |
| 11 | ZnO/SiO_2_ | 30 | 56.5 | 47.3 | 9.20 |
| 12 | ZnO/SiO_2_ | 60 | 100 | 92.5 | 7.50 |

Conditions: PET (1.0 g), catalyst (10 mg), EG (4 g), 180°C.

Table S2. Optimization of catalytic glycolysis of PET.

| Entry | Temp.  (°C) | t  (min) | m_cat_/m_PET_  (mg/g) | PET  conv. (%) | BHET  yield (%) | Oligomer  yield (%) |
| --- | --- | --- | --- | --- | --- | --- |
| 1 | 180 | 10 | 10/1 | 16.8 | 6.70 | 10.1 |
| 2 | 180 | 30 | 10/1 | 56.5 | 47.3 | 9.20 |
| 3 | 180 | 60 | 10/1 | 100 | 92.5 | 7.50 |
| 4 | 180 | 120 | 10/1 | 100 | 92.9 | 7.10 |
| 5 | 180 | 60 | 1/1 | 89.5 | 76.3 | 13.2 |
| 6 | 180 | 60 | 2/1 | 96.5 | 83.2 | 13.3 |
| 7 | 180 | 60 | 10/1 | 100 | 92.5 | 7.50 |
| 8 | 180 | 60 | 20/1 | 100 | 93.2 | 6.80 |
| 9 | 160 | 60 | 10/1 | 46.9 | 36.7 | 10.2 |
| 10 | 170 | 60 | 10/1 | 83.8 | 71.5 | 12.3 |
| 11 | 180 | 60 | 10/1 | 100 | 92.5 | 7.50 |
| 12 | 190 | 60 | 10/1 | 100 | 93.3 | 6.70 |
| 13 | 180 | 60 | 10/1 | 100 | 86.2 | 13.8 |
| 14 | 180 | 60 | 10/1 | 100 | 92.4 | 7.6 |

Reaction condition: PET waste (1.0 g), ZnO/SiO_2_ (10 mg), EG (2 g, 6 g), temperature (180°C), time (1 h).

Table S3. Comparison of reported catalysts for PET glycolysis.

|  | Catalyst | Temp.  (°C) | t  (min) | m_cat_  (g) | BHET  yield (%) | STY  g_BHET_ g_cat_^-1^ h^-1^ |
| --- | --- | --- | --- | --- | --- | --- |
| This work | | 180 | 60 | 0.01 | 92.5 | 122.3 |
|  |  | 180 | 60 | 0.001 | 76.3 | 1009.3 |
| Same glycolysis temperature | Fe/ZnO ^[1]^ | 180 | 40 | 0.002 | 95.5 | 960.9 |
|  | Solarthermal-Co SSCs ^[2]^ | 180 | 180 | 0.00054 | 82.5 | 675.6 |
|  | SO_4_^2-^/CoZnO-300 ^[3]^ | 180 | 180 | 0.003 | 75 | 109.2 |
|  | SO_4_^2-^-ZnO-TiO_2_-200 ^[4]^ | 180 | 180 | 0.003 | 72.8 | 106.7 |
|  | MAF-6 ^[5]^ | 180 | 240 | 0.05 | 81.7 | 26.9 |
|  | DPZIF-8 ^[6]^ | 180 | 240 | 0.01 | 76.1 | 25.1 |
|  | Ultrasmall cobalt NPs ^[7]^ | 180 | 180 | 0.015 | 77 | 22.6 |
|  | [Ch][OAc] ^[8]^ | 180 | 240 | 0.05 | 85.2 | 5.6 |
|  | Solarthermal-CNT-PDA ^[9]^ | 180 | 240 | 0.05 | 81.7 | 5.4 |
|  | TBD: MSA ^[10]^ | 180 | 150 | 0.2 | 82 | 3.4 |
|  | Si-TBD ^[11]^ | 180 | 120 | 0.25 | 92 | 2.9 |
|  | Fe_3_O_4_@SiO_2_/(mim)FeCl_4_ ^[12]^ | 180 | 100 | 0.155 | 88.5 | 1.2 |
|  | (1,3-DMU)/(Zn(OAc)_2_) ^[13]^ | 180 | 1440 | 0.15 | 100 | 0.3 |
| Same glycolysis temperature | Fe_3_O_4_-MWCNT ^[14]^ | 190 | 20 | 0.05 | 82 | 18.5 |
|  | MOF-74(Mg/Mn) ^[15]^ | 190 | 120 | 0.05 | 100 | 13.2 |
|  | Zn-BDC ^[16]^ | 190 | 240 | 0.05 | 91.8 | 6.0 |
|  | [Bmim][OAc] ^[17]^ | 190 | 240 | 0.03 | 89.8 | 9.8 |
| EG critical temperature | DES@ZIF-8 ^[18]^ | 195 | 25 | 0.004 | 83.2 | 132.08 |
|  | CeO_2_-2.7 nm ^[19]^ | 196 | 20 | 0.0125 | 90.3 | 108.2 |
|  | MgO-Al_2_O_3_ ^[20]^ | 196 | 50 | 0.01 | 81.3 | 85.2 |
|  | Zn(OAc)_2_ ^[21]^ | 196 | 60 | 0.01 | 70 | 84.5 |
|  | *c*-ZIF-8@25SiO_2_ ^[22]^ | 197 | 30 | 0.02 | 63.7 | 84.3 |
|  | ZIF8 ^[23]^ | 197 | 90 | 0.01 | 76.7 | 67.6 |
|  | ZnO/SBA-15 ^[24]^ | 197 | 60 | 0.05 | 91 | 26.4 |
|  | h-BNNs/Fe_3_O_4_ ^[25]^ | 200 | 300 | 0.00067 | 100 | 300 |
|  | CoFe_2_O_4_@ZIF-8/ZIF-67 ^[26]^ | 200 | 60 | 0.01 | 83.4 | 110.3 |
|  | Zn-MCM-41 ^[27]^ | 200 | 120 | 0.05 | 80 | 10.5 |
|  | ZnO-MW ^[28]^ | 210 | 45 | 0.01 | 90 | 158.7 |
| PET melting temperature | ZnMn_2_O_4_ ^[29]^ | 260 | 60 | 0.01 | 92.2 | 92 |
|  | GO-Mn_3_O_4_ ^[30]^ | 300 | 80 | 0.01 | 96.4 | 64.2 |
|  | γ-Fe_2_O_3_ ^[31]^ | 300 | 60 | 0.05 | 90 | 26.4 |

Table S4. Catalytic performance of different polyester plastics and mixed plastics.

| Entry | Plastic-type | t  (min) | PET  conv. (%) | BHET  yield (%) | Oligomer  yield (%) |
| --- | --- | --- | --- | --- | --- |
| 1 | PET/PP/PE | 60 | 100 | 88.9 | 11.1 |
| 2 | PET/PE film | 60 | 100 | 92.5 | 7.5 |
| 3 | Water bottle | 60 | 100 | 92.8 | 7.2 |
| 4 | Dyed bottle | 60 | 100 | 92.1 | 7.9 |
| 5 | Packaging film | 60 | 100 | 90.8 | 9.2 |
| 6 | Packaging box | 60 | 100 | 91.2 | 8.8 |
| 7 | Textile | 60 | 100 | 92.3 | 7.7 |
| 8 | Fiber/Cotton | 60 | 100 | 90.1 | 9.9 |
| 9 | PBT^a^ | 90 | 100 | 92.8 | 7.2 |
| 10 | PTT^a^ | 90 | 100 | 91.9 | 8.1 |
| 11 | PET^b^ | 180 | 100 | 92.3 | 7.7 |

Conditions ^a^: PET (1.0 g), catalyst (10 mg), EG (4 g), 180°C.

The upscaling experiment was conducted in a 500 mL single-neck flask under optimized reaction conditions ^b^: 50 g of PET was reacted with 200 g of ethylene glycol in the presence of 1 wt.% catalyst at 180 °C for 3 h.

**Note 2. Process descriptions for PET glycolysis and re-polymerization in ASPEN.**

The poly-non-random two-liquid (NRTL) physical property method was selected in this simulation to deal with the PET glycolysis and flash tank process.

Case 1 (Figure S10): PET waste was uniformly mixed with ethylene glycol (EG) and catalyst. Subsequently, added into an intermittent reaction kettle (RCSTR). The depolymerization reaction was carried out at 180°C for 1 h. After the reaction was completed, the mixture was separated to remove solid catalysts. The mixed reaction liquid is raised to a temperature above the boiling point of EG, and the EG is separated using a flash tank (FLASH). Hot water is introduced into the reaction solution to ensure complete crystallization of recycled bis(2-hydroxyethyl) terephthalate (*r*-BHET) and separation of oligomers. The oligomers are then removed by filtration. The remaining r-BHET containing liquid phase is then processed through a flash tank, where water is flashed off for further reuse. After cooling, high-purity r-BHET can be obtained. To improve energy efficiency, the EG distillate from the flash tank directly enters the heat exchanger (HEATX1) to fully exchange heat with the low-temperature residual liquid from the solid-liquid separator (SEP1). This innovative approach greatly improves the energy efficiency of the separation process and the utilization efficiency of chemical raw materials in the entire process, thereby improving the overall sustainability of PET recycling.

Case 2 (Figure S11): Terephthalic acid (TPA) is homogeneously mixed with ethylene glycol (EG) and a catalyst, then fed into a batch reactor (RCSTR) to undergo esterification at 190 °C for 1 hour. Upon completion of the reaction, hot water is introduced into the mixture to facilitate the complete crystallization of *r*-BHET and to aid in the separation of oligomeric by-products. The resulting oligomers are removed via filtration. The filtrate, containing dissolved r-BHET, is then subjected to a flash separation unit, where water is vaporized and recovered for reuse. After cooling, high-purity *r*-BHET is obtained from the remaining solution.

**Note 3. Goal and scope of life cycle assessment (LCA).**

In this study, the environmental impact of PET recycling was evaluated using LCA. The pre-treatment processes of PET waste were assumed to be consistent with the established protocols for mechanical recycling. In terms of allocation, our study adopts the “cut-off” rule. This principle delineates the original plastic's lifecycle as distinct and separate from that of the recycled plastic, ensuring an independent evaluation of each lifecycle. These form the basis of our “prospective processes”. The functional unit for this LCA is defined as 1 kg of r-BHET in Aspen Plus.

The impact assessment was carried out using the professional software OpenLCA 1.10.3. The assessment methods were IPCC 2021 for GWP and Cumulative Energy Demand for non-renewable energy use (NREU). “Background process” data were sourced from the database ecoinvent V.3.7.1.

**Table S5.** Goal and scope of LCA.

| Goal | |
| --- | --- |
| Reason and scope | 1. Focus on human health, resource and ecosystem.  2. To assess the global warming potential (GWP) and Non-renewable energy use (NREU) of PET recycling, TPA-based production routes and compare it with published commercial depolymerization protocols in Europe or China (Figure 5 in main text) |
| Audience | Industrial stakeholders, the research community, and the public |
| Application | Provide technical support for polyester plastic carbon emission reduction policies and circular economy |
| Intention to use results in comparative studies | Yes, the results are to be compared and disclosed to the public through this article's publication |
| Scope | |
| Product system | The PET waste glycolysis section with the recycled BHET production is based on EU and CN. |
| Functional unit | 1 kg of recycled BHET. |
| System boundary | Cradle-to-factory gate, See Figure S10 |
| Allocation | PET waste cut-off, all environmental effects are allocated to recycled BHET chips. |
| Assumptions | (1) The pre-treatment discharge of waste PET is consistent with the mechanical method  (2) This system deals with 12, 500 kg waste PET/per hour over 8,000 hours/per year  (3) This system is located in Europe or China  (4) Status-quo technology as of 2020 in the background. |
| Requirements on data and quality | Foreground material and energy consumption data were obtained from simulation in Aspen Plus and the background processes were chosen based on Ecoinvent V.3.7.1 in Open LCA 1.10.3. |
| LCIA methodology | IPCC 2021 for GWP; Cumulative Energy Demand for NREU; |
| Impact categories assessed | 1. Global Warming Potential (GWP, 100a), kg CO_2_ equivalent;  2. Non-renewable energy use (NREU), MJ; |
| Limitations | In addition to the above-mentioned assumptions, the following aspects are not assessed: plant construction and equipment maintenance. |

**Table S6.** NREU values of each raw material of PET glycolysis used in the Open LCA.

| Process | Value | Unit | Location |
| --- | --- | --- | --- |
| Zinc oxide/Silica | 29.51090 | MJ per kg | Global of the world |
| Market for ethanol, without water, in 99.7% solution state, from ethylene | 45.49327 | MJ per kg | Europe |
|  | 52.49459 |  | Rest of the world |
| Ultrapure water production | 0.05960 | MJ per kg | Europe |
|  | 0.07874 |  | Rest of the world |
| Market for tap water | 0.00617 | MJ per kg | Europe without Switzerland |
|  | 0.01534 |  | Rest of the world |
| Market for ethylene glycol | 53.00610 | MJ per kg | Global of the world |
| Market group for electricity, low voltage | 9.15115 | MJ per kWh | Europe without Switzerland |
|  | 10.22484 |  | China |
| Heat production, natural gas, at boiler modulating | 1.13333 | MJ per MJ | Europe without Switzerland |
|  | 1.13628 |  | Rest of the world |
| Transport, freight, lorry >32 metric ton | 1.49960 | MJ per t*km  MJ per kg | Europe |
|  | 45.49327 |  | Rest of the world |

**Table S7.** GWP values of each raw material of PET glycolysis used in the Open LCA.

| Process | Value | Unit | Location |
| --- | --- | --- | --- |
| Zinc oxide/Silica | 4.19567 | kg CO_2-eq_ per kg | Global of the world |
| Market for ethanol, without water, in 99.7% solution state, from ethylene | 1.18008 | kg CO_2-eq_ per kg | Europe |
|  | 1.84898 |  | Rest of the world |
| Ultrapure water production | 0.00300 | kg CO_2-eq_ per kg | Europe |
|  | 0.00606 |  | Rest of the world |
| Market for tap water | 0.00034 | kg CO_2-eq_ per kg | Europe without Switzerland |
|  | 0.00106 |  | Rest of the world |
| Market for ethylene glycol | 2.01648 | kg CO_2-eq_ per kg | Global of the world |
| Market group for electricity, low voltage | 0.41694 | kg CO_2-eq_ per kWh | Europe without Switzerland |
|  | 1.05271 |  | China |
| Heat production, natural gas, at boiler modulating | 0.06964 | kg CO_2-eq_ per MJ | Europe without Switzerland |
|  | 0.07065 |  | Rest of the world |
| Transport, freight, lorry >32 metric ton | 0.08599 | kg CO_2-eq_ per t*km | Europe |
|  | 0.08889 |  | Rest of the world |

**Table S8.** LCA results of case-CN recycling route.

| Country | Impact category | Reference unit | Mechanical shredding | Depolymerization | Total |
| --- | --- | --- | --- | --- | --- |
| CN | Fine particulate matter formation | kg PM2.5 eq | 0.00039 | 0.00034 | 0.00073 |
|  | Fossil resource scarcity | kg oil eq | 0.10014 | 0.35681 | 0.45694 |
|  | Freshwater ecotoxicity | kg 1,4-DCB | 0.00888 | 0.00736 | 0.01624 |
|  | Freshwater eutrophication | kg P eq | 0.00004 | 0.00004 | 0.00008 |
|  | Global warming | kg CO_2_ eq | 0.38048 | 1.05781 | 1.43829 |
|  | Human carcinogenic toxicity | kg 1,4-DCB | 0.01209 | 0.03015 | 0.04223 |
|  | Human non-carcinogenic toxicity | kg 1,4-DCB | 0.14255 | 0.15154 | 0.29408 |
|  | Ionizing radiation | kBq Co-60 eq | 0.00437 | 0.00740 | 0.01177 |
|  | Land use | m^2^a crop eq | 0.00525 | 0.00197 | 0.00722 |
|  | Marine ecotoxicity | kg 1,4-DCB | 0.01164 | 0.01016 | 0.02179 |
|  | Marine eutrophication | kg N eq | 0.00000 | 0.00000 | 0.00001 |
|  | Mineral resource scarcity | kg Cu eq | 0.00023 | 0.00062 | 0.00085 |
|  | Ozone formation, Human health | kg NO_x_ eq | 0.00071 | 0.00061 | 0.00132 |
|  | Ozone formation, Terrestrial ecosystems | kg NO_x_ eq | 0.00072 | 0.00065 | 0.00137 |
|  | Stratospheric ozone depletion | kg CFC11 eq | 0.00000 | 0.00000 | 0.00000 |
|  | Terrestrial acidification | kg SO_2_ eq | 0.00087 | 0.00082 | 0.00169 |
|  | Terrestrial ecotoxicity | kg 1,4-DCB | 0.89517 | 0.61566 | 1.51083 |
|  | Water consumption | m^3^ | 0.00064 | 0.02199 | 0.02263 |

**Table S9.** LCA results of case-EU recycling route.

| Country | Impact category | Reference unit | Mechanical shredding | Depolymerization | Total |
| --- | --- | --- | --- | --- | --- |
| EU | Fine particulate matter formation | kg PM2.5 eq | 0.00018 | 0.00025 | 0.00044 |
|  | Fossil resource scarcity | kg oil eq | 0.08044 | 0.34934 | 0.42979 |
|  | Freshwater ecotoxicity | kg 1,4-DCB | 0.00947 | 0.00679 | 0.01626 |
|  | Freshwater eutrophication | kg P eq | 0.00008 | 0.00004 | 0.00012 |
|  | Global warming | kg CO_2_ eq | 0.25009 | 1.02633 | 1.27642 |
|  | Human carcinogenic toxicity | kg 1,4-DCB | 0.01050 | 0.02424 | 0.03474 |
|  | Human non-carcinogenic toxicity | kg 1,4-DCB | 0.14732 | 0.13594 | 0.28326 |
|  | Ionizing radiation | kBq Co-60 eq | 0.04130 | 0.01300 | 0.05430 |
|  | Land use | m^2^a crop eq | 0.00459 | 0.00168 | 0.00627 |
|  | Marine ecotoxicity | kg 1,4-DCB | 0.01242 | 0.00938 | 0.02180 |
|  | Marine eutrophication | kg N eq | 0.00001 | 0.00000 | 0.00001 |
|  | Mineral resource scarcity | kg Cu eq | 0.00026 | 0.00053 | 0.00079 |
|  | Ozone formation, Human health | kg NO_x_ eq | 0.00027 | 0.00052 | 0.00080 |
|  | Ozone formation, Terrestrial ecosystems | kg NO_x_ eq | 0.00029 | 0.00057 | 0.00085 |
|  | Stratospheric ozone depletion | kg CFC11 eq | 0.00000 | 0.00000 | 0.00000 |
|  | Terrestrial acidification | kg SO_2_ eq | 0.00045 | 0.00073 | 0.00118 |
|  | Terrestrial ecotoxicity | kg 1,4-DCB | 0.86252 | 0.58184 | 1.44436 |
|  | Water consumption | m^3^ | 0.00145 | 0.02215 | 0.02360 |

**Table S10.** Comparative LCA results of case-CN recycling route and virgin BHET route.

| Country | Impact category | Reference unit | Virgin BHET | r-BHET/ Virgin BHET (%) |
| --- | --- | --- | --- | --- |
| CN | Fine particulate matter formation | kg PM2.5 eq | 0.00313 | 23.19648 |
|  | Fossil resource scarcity | kg oil eq | 1.44837 | 31.54885 |
|  | Freshwater ecotoxicity | kg 1,4-DCB | 0.08746 | 18.56419 |
|  | Freshwater eutrophication | kg P eq | 0.00054 | 15.68833 |
|  | Global warming | kg CO_2_ eq | 2.65215 | 54.23123 |
|  | Human carcinogenic toxicity | kg 1,4-DCB | 0.15110 | 27.95122 |
|  | Human non-carcinogenic toxicity | kg 1,4-DCB | 1.69936 | 17.30559 |
|  | Ionizing radiation | kBq Co-60 eq | 0.09467 | 12.43253 |
|  | Land use | m^2^a crop eq | 0.02886 | 25.01563 |
|  | Marine ecotoxicity | kg 1,4-DCB | 0.11429 | 19.06708 |
|  | Marine eutrophication | kg N eq | 0.00007 | 11.80068 |
|  | Mineral resource scarcity | kg Cu eq | 0.00866 | 9.80967 |
|  | Ozone formation, Human health | kg NO_x_ eq | 0.00539 | 24.37992 |
|  | Ozone formation, Terrestrial ecosystems | kg NO_x_ eq | 0.00568 | 24.10587 |
|  | Stratospheric ozone depletion | kg CFC11 eq | 0.00001 | 2.20943 |
|  | Terrestrial acidification | kg SO_2_ eq | 0.00686 | 24.60620 |
|  | Terrestrial ecotoxicity | kg 1,4-DCB | 4.73728 | 31.89230 |
|  | Water consumption | m^3^ | 0.04499 | 50.30933 |

**Table S11.** Comparative LCA results of case-EU recycling route and virgin BHET route.

| Country | Impact category | Reference unit | Virgin PET | r-BHET/ Virgin BHET (%) |
| --- | --- | --- | --- | --- |
| EU | Fine particulate matter formation | kg PM2.5 eq | 0.00311 | 14.01406 |
|  | Fossil resource scarcity | kg oil eq | 1.44615 | 29.71937 |
|  | Freshwater ecotoxicity | kg 1,4-DCB | 0.08716 | 18.65222 |
|  | Freshwater eutrophication | kg P eq | 0.00053 | 22.32881 |
|  | Global warming | kg CO_2_ eq | 2.64341 | 48.28685 |
|  | Human carcinogenic toxicity | kg 1,4-DCB | 0.14828 | 23.43069 |
|  | Human non-carcinogenic toxicity | kg 1,4-DCB | 1.69219 | 16.73905 |
|  | Ionizing radiation | kBq Co-60 eq | 0.09478 | 57.29300 |
|  | Land use | m^2^a crop eq | 0.02874 | 21.82815 |
|  | Marine ecotoxicity | kg 1,4-DCB | 0.11388 | 19.14243 |
|  | Marine eutrophication | kg N eq | 0.00007 | 15.62890 |
|  | Mineral resource scarcity | kg Cu eq | 0.00862 | 9.16265 |
|  | Ozone formation, Human health | kg NO_x_ eq | 0.00537 | 14.87638 |
|  | Ozone formation, Terrestrial ecosystems | kg NO_x_ eq | 0.00566 | 15.07185 |
|  | Stratospheric ozone depletion | kg CFC11 eq | 0.00001 | 2.02168 |
|  | Terrestrial acidification | kg SO_2_ eq | 0.00684 | 17.22507 |
|  | Terrestrial ecotoxicity | kg 1,4-DCB | 4.72598 | 30.56210 |
|  | Water consumption | m^3^ | 0.04502 | 52.41501 |

**Table S12.** LCA results in glycolysis of post-consumer PET bottles, functional unit = 1 kg recycled BHET.

| Section | China | | Europe | |
| --- | --- | --- | --- | --- |
|  | NREU | GWP | NREU | GWP |
| Mechanical shredding | 4.68011 | 0.38048 | 4.44505 | 0.25009 |
| PET glycolysis | 16.40980 | 1.02266 | 16.17275 | 0.99205 |
| Total | 21.08991 | 1.40314 | 20.61780 | 1.24214 |

Units: NREU: MJ/kg recycled BHET; GWP: kg CO_2-eq_/kg recycled BHET.

**Table S13.** LCA results in TPA route to produce BHET (virgin BHET route), functional unit = 1 kg virgin BHET.

| Section | China | | Europe | |
| --- | --- | --- | --- | --- |
|  | NREU | GWP | NREU | GWP |
| Virgin BHET | 69.2606 | 2.54919 | 69.16224 | 2.54065 |

Units: NREU: MJ/kg virgin BHET; GWP: kg CO_2-eq_/kg virgin BHET.

**Table S14.** Material and energy input-output of PET bottles glycolysis, functional unit = 1 kg BHET flakes.

| Items | Quantity | Unit |
| --- | --- | --- |
| Input: | | |
| Waste transparent PET bottles | 1088 | kg |
| Transportation distance | 400 | km |
| Electricity, low voltage | 229.8 | kWh |
| Heat (from natural gas) | 2066.8 | MJ |
| Output: | | |
| By-products (e.g., bottle caps, labels) | 88 | kg |
| PET flakes | 1000 | kg |

**Table S15.** Mass balance of post-consumer PET bottles glycolysis.

| **Para.** | CAT1 | EG | FEED | H-EG1 | H-EG2 | H-L2 | L1 | L2 | L3 |
| --- | --- | --- | --- | --- | --- | --- | --- | --- | --- |
| Temp./°C | 25.00 | 180.00 | 179.73 | 110.00 | 197.62 | 196.00 | 180.00 | 180.00 | 99.89 |
| Press/kPa | 101.33 | 101.33 | 101.33 | 0.01 | 101.33 | 101.33 | 101.33 | 101.33 | 101.33 |
| W-PET (kg/h) | 0.00 | 0.00 | 12500.00 | 0.00 | 0.00 | 0.00 | 0.51 | 0.00 | 0.00 |
| Water (kg/h) | 0.00 | 0.00 | 0.00 | 0.00 | 0.00 | 0.00 | 0.00 | 0.00 | 75247.50 |
| EG (kg/h) | 0.00 | 50000.00 | 50000.00 | 46108.22 | 46108.22 | 46119.08 | 46119.08 | 46119.08 | 10.85 |
| BHET (kg/h) | 0.00 | 0.00 | 0.00 | 76.97 | 76.97 | 15576.74 | 15576.74 | 15576.74 | 15499.76 |
| CAT1 (kg/h) | 125.00 | 0.00 | 125.00 | 0.00 | 0.00 | 0.00 | 125.00 | 0.00 | 0.00 |

CAT1: ZnO/SiO_2_.

**Table S15.** Mass balance of post-consumer PET bottles glycolysis (continued).

| **Para.** | L4 | L5 | L6 | L7 | L-EG | OLIGOMER | PRODUCT | R-BHET | R-CAT1 |
| --- | --- | --- | --- | --- | --- | --- | --- | --- | --- |
| Temp./°C | 99.89 | 100.00 | 25.00 | 4.00 | 197.51 | 99.89 | 110.00 | 4.00 | 180.00 |
| Press/kPa | 101.33 | 88.50 | 101.33 | 101.33 | 101.33 | 101.33 | 0.01 | 101.33 | 101.33 |
| W-PET (kg/h) | 0.00 | 0.00 | 0.00 | 0.00 | 0.00 | 0.00 | 0.00 | 0.00 | 0.51 |
| Water (kg/h) | 75247.50 | 7729.49 | 7729.49 | 7729.49 | 0.00 | 0.00 | 0.00 | 0.00 | 0.00 |
| EG (kg/h) | 10.85 | 9.15 | 9.15 | 9.15 | 46108.22 | 0.00 | 10.85 | 0.00 | 0.00 |
| BHET (kg/h) | 15499.76 | 15499.76 | 15499.76 | 15499.76 | 76.97 | 0.00 | 15499.76 | 15499.76 | 0.00 |
| CAT1 (kg/h) | 0.00 | 0.00 | 0.00 | 0.00 | 0.00 | 0.00 | 0.00 | 0.00 | 125.00 |

CAT1: ZnO/SiO_2_.

**Table S15.** Mass balance of post-consumer PET bottles glycolysis (continued).

| **Para.** | R-EG | R-WATER | R-WATER1 | R-WATER2 | W-PET | WATER |
| --- | --- | --- | --- | --- | --- | --- |
| Temp./°C | 197.51 | 100.03 | 100.00 | 4.00 | 25.00 | 99.00 |
| Press/kPa | 101.33 | 101.33 | 88.50 | 101.33 | 101.33 | 101.33 |
| W-PET (kg/h) | 0.00 | 0.00 | 0.00 | 0.00 | 12500.00 | 0.00 |
| Water (kg/h) | 0.00 | 75247.50 | 67518.01 | 7729.49 | 0.00 | 75247.50 |
| EG (kg/h) | 46108.22 | 10.85 | 1.71 | 9.15 | 0.00 | 0.00 |
| BHET (kg/h) | 76.97 | 0.00 | 0.00 | 0.00 | 0.00 | 0.00 |
| CAT1 (kg/h) | 0.00 | 0.00 | 0.00 | 0.00 | 0.00 | 0.00 |

CAT1: ZnO/SiO_2_.

**Table S16.** Mass balance of virgin BHET route.

| **Para.** | BHET | CAT | EG | MIXTURE1 | MIXTURE2 | MIXTURE3 |
| --- | --- | --- | --- | --- | --- | --- |
| Temp./°C | 195.00 | 25.00 | 25.00 | 25.00 | 195.00 | 130.80 |
| Press/kPa | 8.11 | 101.33 | 101.33 | 101.33 | 111.46 | 101.33 |
| Water (kg/h) | 4.83 | 0.00 | 0.00 | 0.00 | 2196.93 | 870.00 |
| EG (kg/h) | 104.26 | 0.00 | 9080.17 | 9080.17 | 1511.05 | 1435.24 |
| CAT2 (kg/h) | 3.04 | 3.04 | 0.00 | 3.04 | 3.04 | 3.04 |
| BHET (kg/h) | 15480.96 | 0.00 | 0.00 | 0.00 | 15501.99 | 15501.99 |
| TPA (kg/h) | 0.00 | 0.00 | 0.00 | 10129.80 | 0.00 | 0.00 |

CAT2: Co(OAc)_2_.

**Table S16.** Mass balance of virgin BHET route (continued).

| **Para.** | R-EG | R-H2O | TPA | VAP1 | VAP2 | VAP3 |
| --- | --- | --- | --- | --- | --- | --- |
| Temp./°C | 177.45 | 100.02 | 25.00 | 130.80 | 195.00 | 171.80 |
| Press/kPa | 101.33 | 101.33 | 101.33 | 101.33 | 8.11 | 101.33 |
| Water (kg/h) | 23.74 | 2168.36 | 0.00 | 1326.92 | 865.17 | 2192.10 |
| EG (kg/h) | 1406.80 | 0.00 | 0.00 | 75.81 | 1330.99 | 1406.80 |
| CAT2 (kg/h) | 0.00 | 0.00 | 0.00 | 0.00 | 0.00 | 0.00 |
| BHET (kg/h) | 21.03 | 0.00 | 0.00 | 0.00 | 21.03 | 21.03 |
| TPA (kg/h) | 23.74 | 2168.36 | 0.00 | 1326.92 | 865.17 | 2192.10 |

CAT2: Co(OAc)_2_.

**Table S17.** Details of the utilities with post-consumer PET bottles glycolysis and virgin BHET route.

| **Unit processes used** | Type of utility used | | | Initial and final state of utility | | |
| --- | --- | --- | --- | --- | --- | --- |
|  |  | | Initial | | | Final |
| COOLING | Water | 20°C; 1.00 atm; liquid phase | | | 24°C; 1.00 atm; liquid phase | |
| L-STEAM | Low-pressure steam | 125°C; 2.29 atm; gaseous phase | | | 124°C; 2.22 atm; gaseous phase | |
| M-STEAM | Medium-pressure steam | 190°C; 8.86 atm; gaseous phase | | | 189°C; 8.65 atm; gaseous phase | |
| H-STEAM | High-pressure steam | 250°C; 84.80 atm; gaseous phase | | | 249°C; 83.67 atm; gaseous phase | |
| ELECT | Electricity |  | | |  | |

**Table S18.** Utility of post-consumer PET bottles glycolysis.

| **Unit processes used** | Type of utility used | Quantity | Function |
| --- | --- | --- | --- |
| RCSTR1 | High pressure steam | 768.03 kg/h | Heating |
| FLASH1 | High pressure steam | 17969.50 kg/h | Heating |
| FLASH2 | Low pressure steam | 69848.66 kg/h | Heating |
| COOLER1 | Water | 263600.46 kg/h | Cooling |
| COOLER2 | Electricity | 323.20 kWh | Cooling |

**Table S19.** Utility of virgin BHET route.

| **Unit processes used** | Type of utility used | Quantity | Function |
| --- | --- | --- | --- |
| RCSTR1 | High pressure steam | 2676.44 kg/h | Heating |
| FLASH2 | High pressure steam | 3146.16 kg/h | Heating |
| RADFRAC1 | Water | 163921 kg/h | Cool the top fraction |
|  | High pressure steam | 1075.2 kg/h | Heat the tower kettle |

**Table S20.** Energy balance of post-consumer PET bottles glycolysis.

| **Equipment** | RCSTR1 | FLASH1 | COOLER1 | COOLER2 | FLASH2 |
| --- | --- | --- | --- | --- | --- |
| **Cost (MJ/h)** | 1320.50 | 30895.43 | -4402.55 | -1163.51 | 153099.82 |

**Table S21.** Energy balance of virgin BHET route.

| **Equipment** | RCSTR1 | FLASH2 | RADFRAC1 | | |
| --- | --- | --- | --- | --- | --- |
|  |  |  | Condenser | Reboiler | |
| **Cost (MJ/h)** | 4601.67 | 5409.27 | -3421.97 | | 1848.61 |

**Note 4. Techno-economic analysis (TEA) assumptions**

To investigate the economic feasibility of this process for PET recycling, we carried out a simplified techno-economic analysis using a model adapted from that of Aspen simulation. The processing capacity of the plant is 100,000 tons of waste PET per year. Table S22 summarize the model used to calculate the plant-gate levelized cost of processing PET. The prices of input chemicals and products are listed in the Tables S22-34.

**List of assumptions made for the calculations:**

1. The capacity factor is expected to be operational on any given day and is assumed to be 0.9, which means the plant will be operational 21.9 hours per day.

2. In the depolymerization stage of waste PET, input chemicals include PET, catalyst, EG, and water. The output products include *r*-BHET and EG.

3. The capital costs of glycolysis and distillation equipment are dependent on the recycling of PET.

4. In this case, the total catalyst cost is just the initial cost of feeding, and the subsequent cycle process is almost negligible due to its low loading, short residence time, and efficient separation of the heterogeneous catalyst.

5. Both operation and maintenance costs are based on actual usage and unit price.

6. Utility cost is based on the unit price of public works simulated by aspen software.

7 Transportation cost is based on shipping distance and price.

8. The clean PET flakes could be obtained at $0.66/kg based on the average mixed bale PET cost in recent years. The price of PET waste ($0.39/kg) was assumed to be 40% of virgin PET. (<https://jiage.molbase.cn/hangqing/PET>). The PET waste textiles could be obtained at $0.13/kg (<https://www.chinacace.org/news/view?id=14859>).

**Table S22.** Total equipment cost of PET recycling.

| **Items** | **Equipment Cost [$]** |
| --- | --- |
| Grinder | 8000 |
| COOLER2 | 22300 |
| HEATX1 | 31400 |
| FLASH1 | 1404200 |
| FLASH2 | 44400 |
| 1-Sep | 16500 |
| 3-Sep | 19100 |
| RCSTR1 | 520500 |
| COOLER1 | 22300 |
| 2-Sep | 31200 |
| Total | 2119900 |

**Table S23.** Total equipment cost of virgin BHET-CN route.

| **Items** | **Equipment Cost [$]** |
| --- | --- |
| RCSTR1 | 535400 |
| RADFRAC1 | 172300 |
| FLASH2 | 20100 |
| FLASH1 | 20100 |
| Total | 747900 |

**Table S24.** Total direct cost, total indirect cost, and total capital investment cost of clean PET, PET wastes, and textile waste recycling.

| Items | Notes | Value [$] |
| --- | --- | --- |
| Equipment cost | The cost of equipment | 2,119,900 |
| Equipment installation | 0.47 * Equipment cost | 996,353 |
| Instrumentation and control | 0.36 * Equipment cost | 763,164 |
| Piping | 0.68 * Equipment cost | 1,441,532 |
| Electrical/heating installation | 0.19 * Equipment cost | 402,781 |
| Utility | Buildings construction + Site development + Service facilities + Delivery | 1,907,910 |
| **Total Direct cost (TDC)** | **Sum of above items** | **7,631,640** |
| Engineering and supervision | 0.10 * TDC | 763,164 |
| Construction fee | 0.10 * TDC | 763,164 |
| Legal expenses | 0.03 * TDC | 228,949 |
| Project Contingency | 0.10 * TDC | 76,316 |
| Construction labor cost | 0.15 * TDC | 1,144,746 |
| Other Cost (Start-Up, Permits) | 0.10 * TDC | 763,164 |
| **Total Indirect Cost (TIC)** | **Sum of above items** | **3,739,504** |
| Fixed Capital Investment (FCI) | TDC+TIC | 11,371,144 |
| Land | 0.10 * FCI | 1,137,114 |
| Working Capital | 0.05 * FCI | 568,557 |
| **Total Capital Investment (TCI)** | **FCI + Land + Working capital** | **13,076,815** |

**Table S25.** Variable operating cost, fix operating cost, general expenses and total raw materials of clean PET, PET wastes, and textile waste recycling.

| Items | Notes | Value [$] | | |
| --- | --- | --- | --- | --- |
| Operating labor | Operating hours * hourly wage * workers | 900,000 | | |
| Operating supervision | 0.15 * Operating labor | 135,000 | | |
| Maintenance and repair | 0.06 * FCI | 682,269 | | |
| Operating supplies | 0.15 * Maintenance and repair | 102,340 | | |
| Laboratory charges | 0.15 * Operating labor | 20,250 | | |
| Royalties | 0.03 * Total product cost | 2,605,863 | 1,768,481 | 869,070 |
| **Variable operating cost** | **Sum of above items** | **4,445,722** | **3,608,340** | **2,708,929** |
| Property tax | 0.02 * FCI | 227,423 | | |
| Insurance | 0.01 * FCI | 113,711 | | |
| Rent | 0.08 * FCI | 909,691 | | |
| Depreciation | 0.20 * FCI | 2,274,229 | | |
| **Fix operating cost** | **Sum of above items** | **3,525,055** | | |
| Administration | 0.20 * Operating labor | 180,000 | | |
| Distribution and selling Research and development | 0.05 * (Variable operating costs + Fix operating costs) | 398,539 | 356,670 | 311,699 |
| Research and development | 0.04 * (Variable operating costs + Fix operating costs) | 318,831 | 285,336 | 249,359 |
| **General expenses** | **Sum of above items** | **897,370** | **822,005** | **741,059** |
| Raw materials | Clean PET, PET waste, textile waste | 66,000,000 | 39,000,000 | 10,000,000 |
| Chemicals | Catalyst | 2,790,000 | | |
| Tap water | For cooling | 1,146,431 | | |
| Steam | As an energy carrier, for heating | 3,716,268 | | |
| Electricity | For all the process using electricity | 1,781,260 | | |
| Transportation | For transporting waste raw materials | 2,560,000 | | |
| **Total raw materials** | **Sum of above items** | **77,993,960** | **50,993,960** | **21,993,960** |

**Table S26.** Total direct cost, total indirect cost, and total capital investment cost of TPA route to produce BHET.

| Items | Notes | Value [$] |
| --- | --- | --- |
| Equipment cost | The cost of equipment | 747,900 |
| Equipment installation | 0.47 * Equipment cost | 351,513 |
| Instrumentation and control | 0.36 * Equipment cost | 269,244 |
| Piping | 0.68 * Equipment cost | 508,572 |
| Electrical/heating installation | 0.19 * Equipment cost | 142,101 |
| Utility | Buildings construction + Site development + Service facilities + Delivery | 673,110 |
| **Total Direct cost (TDC)** | **Sum of above items** | **2,692,440** |
| Engineering and supervision | 0.10 * TDC | 269,244 |
| Construction fee | 0.10 * TDC | 269,244 |
| Legal expenses | 0.03 * TDC | 80,773 |
| Project Contingency | 0.10 * TDC | 26,924 |
| Construction labor cost | 0.15 * TDC | 403,866 |
| Other Cost (Start-Up, Permits) | 0.10 * TDC | 269,244 |
| **Total Indirect Cost (TIC)** | **Sum of above items** | **1,319,296** |
| Fixed Capital Investment (FCI) | TDC+TIC | 4,011,736 |
| Land | 0.10 * FCI | 401,174 |
| Working Capital | 0.05 * FCI | 200,587 |
| **Total Capital Investment (TCI)** | **FCI + Land + Working capital** | **4,613,496** |

**Table S27.** Variable operating cost, fix operating cost, general expenses of TPA route to produce BHET.

| Items | Notes | Value [$] |
| --- | --- | --- |
| Operating labor | Operating hours * hourly wage * workers | 900,000 |
| Operating supervision | 0.15 * Operating labor | 135,000 |
| Maintenance and repair | 0.06 * FCI | 240,704 |
| Operating supplies | 0.15 * Maintenance and repair | 36,106 |
| Laboratory charges | 0.15 * Operating labor | 20,250 |
| Royalties | 0.03 * Total product cost | 2,605,863 |
| **Variable operating cost** | **Sum of above items** | **3,937,923** |
| Property tax | 0.02 * FCI | 80,235 |
| Insurance | 0.01 * FCI | 40,117 |
| Rent | 0.08 * FCI | 320,939 |
| Depreciation | 0.20 * FCI | 802,347 |
| **Fix operating cost** | **Sum of above items** | **1,243,638** |
| Administration | 0.20 * Operating labor | 180,000 |
| Distribution and selling Research and development | 0.05 * (Variable operating costs + Fix operating costs) | 259,078 |
| Research and development | 0.04 * (Variable operating costs + Fix operating costs) | 207,262 |
| **General expenses** | **Sum of above items** | **646,340** |
| Chemicals | TPA, EG, catalyst | 171,164,619 |
| Tap water | For cooling | 563,888 |
| Steam | As an energy carrier, for heating | 208,728 |
| **Total raw materials** | **Sum of above items** | **171,937,235** |

**Table S28.** Total direct cost, total indirect cost, land, working capital and total capital investment cost of PET waste recycling.

| **Items** | **Value (Million $)** |
| --- | --- |
| Total Direct cost (TDC) | 7.63 |
| Total Indirect Cost (TIC) | 3.74 |
| Land | 1.14 |
| Working Capital | 0.57 |
| Total Capital Investment (TCI) | 13.08 |

**Table S29.** Total direct cost, total indirect cost, land, working capital and total capital investment cost of TPA route to produce BHET.

| **Items** | **Value (Million $)** |
| --- | --- |
| Total Direct cost (TDC) | 2.69 |
| Total Indirect Cost (TIC) | 1.32 |
| Land | 0.40 |
| Working Capital | 0.20 |
| Total Capital Investment (TCI) | 4.61 |

**Table S30.** Utility operation cost of clean PET recycling.

| **Items** | **Value ($/year)** | **Yield (t/year)** | **Unit production cost ($/t)** |
| --- | --- | --- | --- |
| Variable operating cost | 4,445,722 | 123,998 | 35.9 |
| Fix operating cost | 3,525,055 |  | 28.4 |
| General expenses | 897,370 |  | 7.2 |
| Total raw materials | 77,993,960 |  | 629.0 |
| Total production cost | 86,862,106 |  | 700.5 |

**Table S31.** Utility operation cost of PET waste recycling.

| **Items** | **Value ($/year)** | **Yield (t/year)** | **Unit production cost ($/t)** |
| --- | --- | --- | --- |
| Variable operating cost | 360,8340 | 123,998 | 29.1 |
| Fix operating cost | 3,525,055 |  | 28.4 |
| General expenses | 822,005 |  | 6.6 |
| Total raw materials | 50,993,960 |  | 411.3 |
| Total production cost | 58,949,359 |  | 475.4 |

**Table S32.** Utility operation cost of textile waste recycling.

| **Items** | **Value ($/year)** | **Yield (t/year)** | **Unit production cost ($/t)** |
| --- | --- | --- | --- |
| Variable operating cost | 2,708,929 | 123,998 | 21.8 |
| Fix operating cost | 3,525,055 |  | 28.4 |
| General expenses | 741,059 |  | 6.0 |
| Total raw materials | 21,993,960 |  | 177.4 |
| Total production cost | 28,969,002 |  | 233.6 |

**Table S33.** Utility operation cost of TPA route to produce BHET.

| **Items** | **Value ($/year)** | **Yield (t/year)** | **Unit production cost ($/t)** |
| --- | --- | --- | --- |
| Variable operating cost | 3,937,923 | 123,848 | 31.8 |
| Fix operating cost | 1,243,638 |  | 10.0 |
| General expenses | 646,340 |  | 5.2 |
| Total raw materials | 171,937,235 |  | 1388.3 |
| Total production cost | 177,765,137 |  | 1435.3 |

**Table S34.** MSP of PET recycling, TPA route to produce BHET, and literature.

| **Items** | **Value ($/t)** |
| --- | --- |
| PET recycling | 700.5 (clean PET) |
|  | 475.4 (PET waste) |
|  | 233.6 (textile waste) |
| TPA route to produce BHET | 1435.3 |
| ZnO-MW^[32]^ | 1495.7 |
| GB-richCeO_2_^[33]^ | 1290 |
| Cu_1_Ru_1_ superstructures^[34]^ | 1020 |
| CoMn_2_O_4_^[35]^ | 1312 |
| Spent LiCoO_2_^[36]^ | 1679 |

## Renferences:

[1] J. Cao, Y. Lin, W. Jiang, W. Wang, X. Li, T. Zhou, P. Sun, B. Pan, A. Li, Q. Zhang, *ACS Sustain. Chem. Eng.* **2022**, *10*, 5476-5488.

[2] Y. Liu, X. Wang, Q. Li, T. Yan, X. Lou, C. Zhang, M. Cao, L. Zhang, T. K. Sham, Q. Zhang, L. He, J. Chen, *Adv. Funct. Mater.* **2022**, *33*, 2210283.

[3] M. Zhu, Z. Li, Q. Wang, X. Zhou, X. Lu, *Ind. Eng. Chem. Res.* **2012**, *51*, 11659-11666.

[4] M. Zhu, S. Li, Z. Li, X. Lu, S. Zhang, *Chem. Eng. J.* **2012**, *185-186*, 168-177.

[5] R.-X. Yang, Y.-T. Bieh, C. H. Chen, C.-Y. Hsu, Y. Kato, H. Yamamoto, C.-K. Tsung, K. C. W. Wu, *ACS Sustain. Chem. Eng.* **2021**, *9*, 6541-6550.

[6] N. Han, K. Lee, J. Lee, J. H. Jo, E. J. An, G. Lee, W. S. Chi, C. Lee, *Chemosphere* **2024**, *364*, 143187.

[7] F. R. Veregue, C. T. Pereira da Silva, M. P. Moisés, J. G. Meneguin, M. R. Guilherme, P. A. Arroyo, S. L. Favaro, E. Radovanovic, E. M. Girotto, A. W. Rinaldi, *ACS Sustain. Chem. Eng.* **2018**, *6*, 12017-12024.

[8] Y. Liu, X. Yao, H. Yao, Q. Zhou, J. Xin, X. Lu, S. Zhang, *Green Chem.* **2020**, *22*, 3122-3131.

[9] Y. Liu, Q. Zhong, P. Xu, H. Huang, F. Yang, M. Cao, L. He, Q. Zhang, J. Chen, *Matter* **2022**, *5*, 1305-1317.

[10] C. Jehanno, I. Flores, A. P. Dove, A. J. Müller, F. Ruipérez, H. Sardon, *Green Chem.* **2018**, *20*, 1205-1212.

[11] Fehér, J. Kiss, P. Kisszékelyi, J. Molnár, P. Huszthy, L. Kárpáti, J. Kupai, *Green Chem.* **2022**, *24*, 8447-8459.

[12] Cano, C. Martin, J. A. Fernandes, R. W. Lodge, J. Dupont, F. A. Casado-Carmona, R. Lucena, S. Cardenas, V. Sans, I. de Pedro, *Appl. Catal. B Environ. 260, 118110 (2020)*.

[13] B. Liu, W. Fu, X. Lu, Q. Zhou, S. Zhang, *ACS Sustain. Chem. Eng.* **2018**, *7*, 3292-3300.

[14] A. M. Al-Sabagh, F. Z. Yehia, D. R. K. Harding, G. Eshaq, A. E. ElMetwally, *Green Chem.* **2016**, *18*, 3997-4003.

[15] M. A. Baluk, P. J. Trzebiatowska, A. Pieczyńska, D. Makowski, M. Kroczewska, J. Łuczak, A. Zaleska-Medynska, *J. Environ. Manage.* **2024**, *363*, 121360.

[16] D. D. Pham, D.-N. Vo, M. T. Phong, H. H. Nguyen, T. Nguyen-Thoi, T.-P. T. Pham, D. Ha Le Phuong, L. K. H. Pham, D. H. Won, D. L. T. Nguyen, T. M. Nguyen, *Chem. Eng. J.* **2024**, *490*, 151667.

[17] A. M. Al-Sabagh, F. Z. Yehia, A.-M. M. F. Eissa, M. E. Moustafa, G. Eshaq, A.-R. M. Rabie, A. E. ElMetwally, *Ind. Eng. Chem. Res.* **2014**, *53*, 18443-18451.

[18] R. Wang, T. Wang, G. Yu, X. Chen, *Polym. Degrad. Stabil.* **2021**, *183*, 109463.

[19] L.-X. Yun, H. Wu, Z.-G. Shen, J.-W. Fu, J.-X. Wang, *ACS Sustain. Chem. Eng.* **2022**, *10*, 5278-5287.

[20] F. Chen, G. Wang, W. Li, F. Yang, *Ind. Eng. Chem. Res.* **2012**, *52*, 565-571.

[21] R. López-Fonseca, I. Duque-Ingunza, B. de Rivas, S. Arnaiz, J. I. Gutiérrez-Ortiz, *Polym. Degrad. Stabil.* **2010**, *95*, 1022-1028.

[22] X. Lou, X. Gao, Y. Liu, M. Chu, C. Zhang, Y. Qiu, W. Yang, M. Cao, G. Wang, Q. Zhang, J. Chen, *Chin. J. Catal.* **2023**, *49*, 113-122.

[23] Q. Suo, J. Zi, Z. Bai, S. Qi, *Catal. Lett.* **2016**, *147*, 240-252.

[24] H. Yao, L. Liu, D. Yan, Q. Zhou, J. Xin, X. Lu, S. Zhang, *Chem. Eng. Sci.* **2022**, *248*, 117109.

[25] M. R. Nabid, Y. Bide, M. Jafari, *Polym. Degrad. Stabil.* **2019**, *169*, 108962.

[26] T. Wang, C. Shen, G. Yu, X. Chen, *Fuel* **2021**, *304*, 121397.

[27] C. Dai, Y. Liu, Z. Wang, G. Yu, *Cata. Today* **2024**, *440*, 114827.

[28] E. Andini, P. Bhalode, E. Gantert, S. Sadula, D. G. Vlachos, *Sci. Adv.* **2024**, *10*, eado6827.

[29] M. Imran, D. H. Kim, W. A. Al-Masry, A. Mahmood, A. Hassan, S. Haider, S. M. Ramay, *Polym. Degrad. Stabil.* **2013**, *98*, 904-915.

[30] G. Park, L. Bartolome, K. G. Lee, S. J. Lee, D. H. Kim, T. J. Park, *Nanoscale* **2012**, *4*, 3879–3885.

[31] L. Bartolome, M. Imran, K. G. Lee, A. Sangalang, J. K. Ahn, D. H. Kim, *Green Chem.* **2014**, *16*, 279-286.

[32] Y. Luo, E. Selvam, D. G. Vlachos, M. Ierapetritou, *ACS Sustain. Chem. Eng.* **2023**, *11*, 4209-4218.

[33] Y. Liu, P. Yan, X. Li, Q. Li, S. Li, H. Han, M. Chu, J. Fu, M. Cao, P. Xu, Q. Zhang, L. He, J. Chen, *Adv. Mater.* **2025**, *37*, e2412740.

[34] M. Chu, P. Yan, Y. Zhou, X. Lou, C. Li, M. Cao, L. He, Q. Zhang, J. Chen, *Adv. Funct. Mater.* **2025**, *35*, 2417644.

[35] J. Ruan, Q. Cao, X. Li, Q. Ren, M. Li, S. Dong, N. Li, Q. Xu, H. Li, J. Lu, D. Chen, *Adv. Mater.* **2025**, *37*, e2500090.

[36] X. Lou, P. Yan, B. Jiao, Q. Li, P. Xu, L. Wang, L. Zhang, M. Cao, G. Wang, Z. Chen, Q. Zhang, J. Chen, *Nat. Commun.* **2024**, *15*, 2730
